# A Model for Genome Size Evolution

**DOI:** 10.1007/s11538-014-9997-8

**Published:** 2014-08-21

**Authors:** Stephan Fischer, Samuel Bernard, Guillaume Beslon, Carole Knibbe

**Affiliations:** 1 INSA-Lyon, Inria, CNRS, LIRIS, UMR5205, 69621 Villeurbanne, France; 2 Inria, Institut Camille Jordan, CNRS, UMR5208, 69622 Villeurbanne, France; 3 Université Lyon 1, Inria, CNRS, LIRIS, UMR5205, 69622 Villeurbanne, France

**Keywords:** Genome size, Molecular evolution, Chromosomal rearrangements, Markov chain, Doeblin’s condition

## Abstract

We present a model for genome size evolution that takes into account both local mutations such as small insertions and small deletions, and large chromosomal rearrangements such as duplications and large deletions. We introduce the possibility of undergoing several mutations within one generation. The model, albeit minimalist, reveals a non-trivial spontaneous dynamics of genome size: in the absence of selection, an arbitrary large part of genomes remains beneath a finite size, even for a duplication rate 2.6-fold higher than the rate of large deletions, and even if there is also a systematic bias toward small insertions compared to small deletions. Specifically, we show that the condition of existence of an asymptotic stationary distribution for genome size non-trivially depends on the rates and mean sizes of the different mutation types. We also give upper bounds for the median and other quantiles of the genome size distribution, and argue that these bounds cannot be overcome by selection. Taken together, our results show that the spontaneous dynamics of genome size naturally prevents it from growing infinitely, even in cases where intuition would suggest an infinite growth. Using quantitative numerical examples, we show that, in practice, a shrinkage bias appears very quickly in genomes undergoing mutation accumulation, even though DNA gains and losses appear to be perfectly symmetrical at first sight. We discuss this spontaneous dynamics in the light of the other evolutionary forces proposed in the literature and argue that it provides them a stability-related size limit below which they can act.

## Introduction

Genome lengths span several order of magnitudes across all living species (Koonin 2008, 2009) and the origin of these variations is still unclear. Total genome size does not correlate well with organismal complexity, a paradox called “C-value paradox” in the 1970s (Thomas 1971). When it was discovered that DNA comprises not only genes but also a lot of non-coding sequences, it felt logical to rather search for a correlation between gene number and organismal complexity. There again, no obvious correlation was found, a phenomenon called the “G-value paradox” (Betrán and Long 2002; Hahn and Wray 2002). One reason could be the difficulty to define an objective and quantitative measure of organismal complexity. But even if such a good measure was available, its correlation with genome size or gene number could very well be low anyway. Indeed, genome size results from a tension between multiple evolutionary pressures, some acting at the mutation level, other at the selection level, some tending to make the genome grow, other tending to make it shrink. It is thus essential to understand how each pressure acts individually in order to disentangle their interactions.

The formalism we propose here sets a general framework for the study of the impact of mutational mechanisms on genome length with two important features: (i) genomes can undergo both small indels and large chromosomal rearrangements and (ii) genomes undergo a size-dependent number of mutations rather than limiting the mutations to one per replication. This framework allows us to give a simple condition for the existence and uniqueness of a stationary distribution for genome size in the absence of selection, and we characterize how each type of mutation impacts the spontaneous dynamics of genome size. We find that for a wide range of mutation rates, the spontaneous dynamics of genome size naturally prevents it from growing indefinitely.

We present the details of the mathematical model and the hypotheses that underlie the biological mechanisms for mutations and replication in Sect. 2. In Sect. 3, we analyze the evolution of genome size in the absence of selection. It shows that a stationary distribution exists even if duplications are twice as frequent as deletions. In Sect. 4, we use a continuous approximation to analyze further the outcome of mutations in a single generation and show that one generation is already enough for genome size to be bounded, independently from the initial sizes of the genomes. In Sect. 5, we generalize the results to various distributions for the size of mutations and to the presence of selection. As the bounds found in Sect. 4 apply for every generation, we argue that selection cannot help overcome the bounds found in Sect. 4 but determines how the population behaves with respect to these bounds. In order to illustrate how our results apply in biologically plausible situations, we propose numerical simulations of genome size evolution in mutation accumulation experiments in Sect. 6. We discuss the extensions and limits of the model, as well as the links with previous studies, in Sect. 7.

## A Model for Genome Size Evolution

### Definition of the Model

We consider four types of mutations that occur in natural genomes: small insertions and deletions (hereafter called indels), large deletions and duplications. In this study, we suppose that the impact of mutations on a genome of size $s_0$ is as follows:

- For small insertions, 1 to $l_\mathrm{{ins}}$ bases are added to the genome. The size after one mutation belongs to $\{s_0+1,\ldots ,s_0+l_\mathrm{{ins}}\}$. The transition probabilities can be defined arbitrarily, but we suppose they do not depend on the starting state $s_0$. The state $s_0=0$ can be escaped through small insertions.
- For small deletions, 1 to $l_\mathrm{{sdel}}$ bases are removed from the genome (if possible). The size after one mutation belongs to $\{\max (0,s_0-l_\mathrm{{sdel}}),\ldots ,\max (0,s_0-1)\}$. The transition probabilities can be defined arbitrarily, but should not depend on the starting size $s_0$. All the transitions that go below 0 are rewired to 0. If $s_0=0$, the size after the small deletion is $0$ with probability 1.
- For duplications, 1 to $s_0$ bases are added to the genome. The size after one mutation belongs to $\{s_0+1,\ldots ,2s_0\}$. We suppose that each final state is reached with probability $1/s_0$. If $s_0=0$, the size after the duplication is $0$ with probability 1.
- For large deletions, 1 to $s_0$ bases are removed from the genome. The size after one mutation belongs to $\{0,\ldots ,s_0-1\}$. We suppose that each final state is reached with probability $1/s_0$. If $s_0=0$, the size after the large deletion is $0$ with probability 1.

We suppose that small deletions (resp. insertions) and large deletions (resp. duplications) occur according to different mechanisms (thus at different rates). The fact that the indel distribution does not depend on $s_0$ is not strictly necessary, but makes one part of the proof simpler (see Remark 4 in Appendix 1). The important assumption is that there is an upper bound on the size of indels ($l_\mathrm{{ins}}$ for small insertions and $l_\mathrm{{sdel}}$ for small deletions), but these bounds may be arbitrarily large (several kb for example). Indels can be thought as representing two kinds of events. First, replication slippage of the DNA polymerase can lead to the loss or gain of a few base pairs. Second, the transposition of transposable elements leads to the gain of up to 10 kb. They can be incorporated as small insertions in our model. However, note that here the insertion rate will be defined per base pair whereas the transposition rate is normally given per transposable element. One could imagine a more complex model where two organization levels are considered: the base pair level for some mutational mechanisms and the copy number level for other elements such as transposable elements or tandemly repeated sequences, but that would increase the number of parameters. By choosing the base pair level and expressing the mutation rate per base pair, the spontaneous rate of transposition will be higher than normally expected. Hence the pressure toward genome growth is high in the model. Therefore, the convergence toward finite sizes proved in this growth-prone model (Theorem 2) should arguably hold in the more realistic model where transposable elements replicate more moderately based on their copy number.

For large deletions and duplications, we have assumed that the number of base pairs that are lost or gained follows a uniform distribution between 1 and the current genome size. Mechanistically, if the two end points of a deletion (or a duplication) are taken at random along the genome, the resulting distribution of losses (or gains) is uniform. We use these distributions as a guideline through the paper but this is not necessary for showing that the genome size remains bounded. We will see that the proof holds for a more general family of distributions (see Sect. 5, Corollary 1). It is also important to note that these distributions reflect the *spontaneous* events. Estimations of the distributions of rearrangements based on *fixed* events (filtered by natural selection) yield exponential or, more generally, gamma distributions (Sankoff et al. 2005; Darling et al. 2008). The spontaneous distributions are generally not accessible because large events are likely to be lethal and thus not observable. Data from bacteria suggest that they could follow a lognormal law (see Sect. 6, Fig. 3).

There is evidence that large events occur in all species. In bacterial strains cultivated in laboratory conditions, amplifications and numerous large deletions through ectopic recombination have been observed, the size of single deletions reaching up to more than 200 kb under weak selection (Porwollik et al. 2004; Nilsson et al. 2005). What is more, at least locally, the deletion sizes might be uniform because of random insertions of transposable elements (Cooper et al. 2001). In the human genome, duplications and large deletions causing genetic diseases have been identified. For example, in half of the cases, the Charcot-Marie-Tooth disease is caused by a 1.4 Mb duplication. Another example is the Smith-Magenis syndrom, often associated with a partial deletion of chromosome 17, spanning from 950 kb to 9 Mb (Lupski 2007). For comparison purposes, a deletion of 9 Mb is approximately twice the size of the whole genome of *E. coli* K12 (4.6 Mb). Additionally, whole chromosomes, or even genomes, can be lost or duplicated because of segregation problems during cell division. Whole genome duplications have been selected frequently through the history of life and numerous genomes bear traces of such events (Jaillon et al. 2009).

For each type of mutation, we define a mutation rate expressed as a number of mutations per base pair per generation: $\mu _\mathrm{{ins}}$ for small insertions, $\mu _\mathrm{{sdel}}$ for small deletions, $\mu _\mathrm{{ldel}}$ for large deletions and $\mu _\mathrm{{dup}}$ for duplications. We call $\mu = \mu _\mathrm{{ins}}+ \mu _\mathrm{{sdel}}+ \mu _\mathrm{{ldel}}+ \mu _\mathrm{{dup}}$ the total mutation rate per base pair per generation (note that in this paper, the term “mutations” refers to small indels and chromosomal rearrangements). For every generation, we suppose that the occurrences of mutations of type $type$ along a genome follow independent Poisson processes with rate $\mu _\mathrm{{type}}$, where $\mu _\mathrm{{type}}$ is the rate of the mutation considered. The total number of mutations per generation is given by a Poisson law with parameter $\mu s_0$, where $s_0$ is the size of the genome considered at the beginning of the generation. As we shall see later (Sect. 5), allowing for several mutations per generation is essential when we include selection in the model. As a result of the independence of the Poisson processes, the probability that any given mutation is a small insertion (for example) is $\mu _\mathrm{{ins}}/\mu$. We can write the mutations as transitions on $\mathbb {N}$, the space of all possible genome sizes. We chose not to have a predefined maximal genome size to ensure that infinite growth is possible and that the convergence to a stationary distribution is not trivial.

We define two transition matrices on this space. The first, called $\mathbf{M}_1$, describes the action of a single mutation. The second matrix, called $\mathbf{M}_G$, gives the transitions for one generation, when all mutations have been drawn according to independent Poisson processes.

#### **Definition 1**

$\mathbf{M}_1= ((\mathbf{M}_1)_{ij})_{i,j \in \mathbb {N}}$ where $(\mathbf{M}_1)_{ij}$ is the probability that a genome having initial size $i$ ends up having size $j$ after *exactly one mutation*. $\mathbf{M}_1$ is a stochastic matrix. The transition rates from state $i$ to state $j$ are computed according to the definitions above. $\mathbf{M}_1$ gives the evolution of genome size mutation after mutation.

#### **Definition 2**

$\mathbf{M}_G= ((\mathbf{M}_G)_{ij})_{i,j \in \mathbb {N}\backslash \{0\}}$ where $(\mathbf{M}_G)_{ij}$ is the probability that a genome having initial size $i$ ends up having size $j$ after *one generation*. Several mutations can occur in one generation depending on the rates $\mu _\mathrm{{sdel}}$, $\mu _\mathrm{{ins}}$, $\mu _\mathrm{{ldel}}$ and $\mu _\mathrm{{dup}}$. $\mathbf{M}_G$ gives the evolution of genome size generation after generation.

Importantly, we define $\mathbf{M}_G$ on $\mathbb {N}^*=\mathbb {N}\backslash \{0\}$ instead of $\mathbb {N}$. All individuals ending up with length 0 after complete replication are automatically reassigned to the state with length 1. While 0 is not an absorbing state in the Markov chain $(\mathbb {N},\mathbf{M}_1)$ because of small insertions, it would be absorbing in the Markov chain $(\mathbb {N},\mathbf{M}_G)$ because the number of mutations per replication given by the Poisson law is 0. Therefore, if we kept this state, it would partially affect the spontaneous dynamics of genomes: even if genomes tended do grow, there would be a nonzero probability that they remain trapped in the absorbing state. In $(\mathbb {N}^*,\mathbf{M}_G)$, we made sure that there is no absorbing state (there is a nonzero probability to leave every state), thus no trivial stationary distribution.

We define a population vector $\varvec{\nu }_t$ such that $\forall t \in \mathbb {N}$, $\varvec{\nu }_t$ is a probability measure on $\mathbb {N}^*$, corresponding to the density of an infinite population. $\varvec{\nu }_t(s)$ represents the fraction of genomes with size $s$ at generation $t$. We consider an arbitrary starting population $\varvec{\nu }_0$. In the special case where all genome states confer the same probability of reproduction (no selection), the evolution of the population is given by

1 $$\begin{aligned} \varvec{\nu }_{t+1} = \varvec{\nu }_t \mathbf{M}_G\end{aligned}$$

Because $\mathbf{M}_G$ is stochastic and does not depend on $t$, Eq. (1) can be interpreted as describing the evolution of the time-homogeneous Markov chain $(\mathbb {N}^*,\, \mathbf{M}_G)$ in the space of genome sizes.

### Fundamental Properties of the Gain and Loss Distributions

Because mutations will accumulate with time (within a generation or along a lineage), it is essential to understand how the effects of these mutations on genome size will “add up” in order to understand whether genomes have a tendency to grow or to shrink. The model includes processes of different nature. Small indels have additive effects and the average impact of an indel does not depend on the initial genome size. For equal rates of small insertions and small deletions $(\mu _\mathrm{{ins}}=\mu _\mathrm{{sdel}})$ and for the same length distribution of insertions and small deletions $(l_\mathrm{{ins}}=l_\mathrm{{sdel}})$ in particular, the transitions due to small indels are symmetrical. On the contrary, duplications and large deletions have a multiplicative effect on genome size and the average gains and losses vary with the starting genome size $s_0$. Explicitly, the average gains and losses are

2 $$\begin{aligned} \frac{1+2+\cdots +s_0}{s_0}= \frac{s_0(s_0+1)}{2s_0} \simeq \frac{s_0}{2}. \end{aligned}$$

**Fig. 1:**
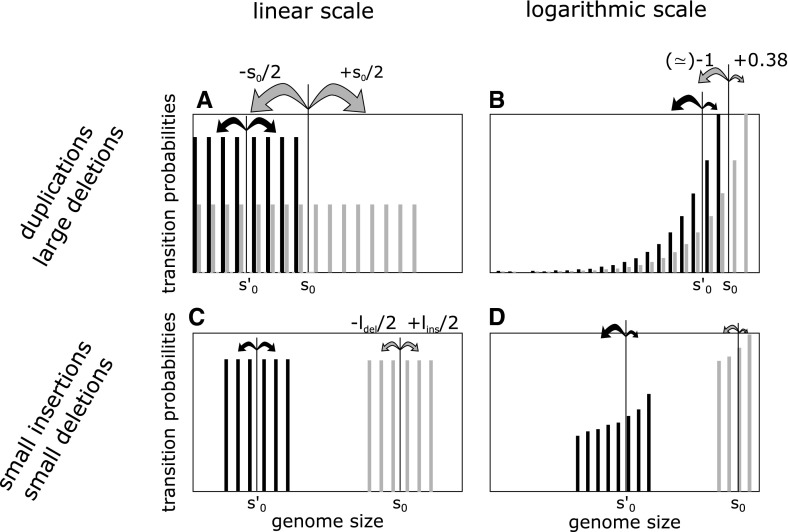
Schematic densities of transitions in linear and logarithmic scales for two different starting states $s_0$ (schematic transition density in *gray*) and $s'_0$ (schematic transition density in *black*). The *arrows* indicate the size of the average jumps for each type of mutation. **a** In linear scale, for equal rates, the duplication and large deletion processes look symmetrical, but the average jumps depend on the starting point. **b** In logarithmic scale, the apparent symmetry is broken: there is a clear tendency to shrink and the average jumps become nearly equal for every starting point. **c** The linear scale is perfectly adapted for indels that occur at equal rates when the distribution does not depend on the starting size. **d** The logarithmic scales breaks the symmetrical properties of indels, but their impact becomes smaller with the initial size

As illustrated in Fig. 1a, for equal duplication and large deletion rates, the transitions might look symmetrical because for a given starting point, gains and losses compensate each other. However, this does not give a good indication for our process, as in fact we need to know whether a loss or a gain that was just undergone will be compensated, taking into account the fact that *genome size has changed* between the two mutations. In linear scale, this question is difficult to answer because average gains and losses keep changing. Indeed, as depicted on Fig. 1a, a smaller genome undergoes smaller average gains and losses. For example, if a genome undergoes a deletion followed by a duplication, the loss will be on average bigger than the gain, as the genome will have reached a smaller size between the two mutations. This remains true if it undergoes the duplication first, so we expect an average loss, even if the distributions are symmetrical and happen at the same rate. Hence, the overall average change in genome size is difficult to predict, as it is the sum of ever-changing average gains and losses.

In order to aggregate losses and gains efficiently, we need to find a scale in which the average impacts of deletions and duplications do not change with genome size, so we can simply add them up without worrying about intermediate states. This is the case in logarithmic scale (Fig. 1b), in which the gain and loss distributions become nearly invariant by translation. It becomes clear that the duplication/large deletion process is *de facto* biased toward shrinkage as, on average, approximately 2.59 duplications are needed to revert a deletion (see Property 1 below). For indels, the linear scale is well-adapted (Fig. 1c) but, as they are asymptotically negligible compared to duplications and large deletions (Fig. 1d), we choose logarithmic scale over linear scale.

#### **Definition 3**

We call $S_n$ the random variable giving the state of $(\mathbb {N},\mathbf{M}_1)$ after $n$ mutations. In probability notation, the starting point $s_0 \in \mathbb {N}$ is written as a subscript, as in $\mathrm{Pr}_{s_0}\left[ S_n = k\right] = (\mathbf{M}_1^n)_{s_0k}$, the probability that the size $k \in \mathbb {N}$ is reached in $n$ mutations, starting from $s_0$. For simplicity, when the starting size $s_0$ has no influence, we drop the subscript, as in $\mathrm{Pr}_{s_0}\left[ S_{n+1} = j | S_n = i \right] = \mathrm{Pr}_{}\left[ S_{n+1} = j | S_n = i \right] = (\mathbf{M}_1)_{ij}$.

#### **Property 1**

Let $\Delta (s)= \mathbb {E}_{}\left[ \log (S_{n+1})|S_n = s\right] -\mathbb {E}_{}\left[ \log (S_{n})|S_n = s\right]$, the average size of one-mutation jumps in logarithmic scale, starting from $s$.

- if the (n+1)th mutation is a large deletion, $\Delta (s) \underset{s\rightarrow +\infty }{\longrightarrow } -1$.
- if the (n+1)th mutation is a duplication, $\Delta (s) \underset{s\rightarrow +\infty }{\longrightarrow } 2\log 2-1$.
- if the (n+1)th mutation is an indel, $\Delta (s) \underset{s\rightarrow +\infty }{\longrightarrow } 0$.

The proof of this property is given at the beginning of Appendix 1 (restated as Property 5).

## Existence and Uniqueness of a Stationary Distribution for the Generational Markov Chain $(\mathbb {N}^*,\mathbf{M}_G)$

Equation (1) corresponds to the Markov chain $(\mathbb {N}^*,\mathbf{M}_G)$, it describes the evolution of genome size in the absence of selection. We will show the existence and uniqueness of a stationary distribution for genome size using the following extension of Doeblin’s condition.

### **Theorem 1**

(Doeblin’s condition in $g$ steps) Let $\mathbf{M}$ be a transition probability matrix on a state space $\mathbb {S}$ with the property that, for some integer $g \ge 1$, some state $i_f \in \mathbb {S}$ and $\varepsilon > 0,\, (\mathbf{M}^g)_{ii_f} \ge \varepsilon$ for all $i \in \mathbb {S}$. Then $\mathbf{M}$ has a unique stationary probability vector $\varvec{\pi },\, (\varvec{\pi })_{i_f} \ge \varepsilon$. In other words, for all initial distributions $\varvec{\mu }$, the system converges to the distribution given by $\pi$. Mathematically,

$$\begin{aligned} \Vert \varvec{\mu } \mathbf{M}^t - \varvec{\pi } \Vert \le 2(1 - \varepsilon )^{\lfloor \frac{t}{g}\rfloor }, \quad t \ge 0. \end{aligned}$$

In this definition and in the rest of this article, the norm is the 1-norm, e.g., $\Vert \varvec{\mu }\Vert = \sum _{i \in X} \left| \varvec{\mu }_i \right|$. A proof of this theorem can be found in Stroock (2005). In this section, we will use Doeblin’s condition in $g=2$ steps (generations) to prove the following theorem.

### **Theorem 2**

(Stationary distribution for genome size without selection) If $(2\log 2-1) {\mu _\mathrm{{dup}}} < {\mu _\mathrm{{ldel}}}$, then the Markov chain $(\mathbb {N}^*,\mathbf{M}_G)$ has a unique asymptotic stationary probability vector $\varvec{\nu }_{\infty }$. For any initial distribution $\varvec{\nu }_0$, the distribution of genome sizes converges to $\varvec{\nu }_{\infty }$. Mathematically,

$$\begin{aligned} \lim _{t\rightarrow \infty } \Vert \varvec{\nu }_0 \mathbf{M}_G^t - \varvec{\nu }_\infty \Vert = 0 \end{aligned}$$

Biologically, the convergence of the distribution implies that, even after a long time of evolution, genome size does not tend to infinity: an arbitrary large part of genomes is located beneath a finite size. The rate of small insertions and small deletions does not impend the convergence of the system. In particular, the rate of transposition of transposable elements can be arbitrarily large, genomes will still converge toward finite sizes. What is more, genome size remains finite for a duplication rate $\mu _\mathrm{{dup}}$ as large as ${\simeq }2.6$ higher than the rate of large deletions $\mu _\mathrm{{ldel}}$.

The remainder of this section is dedicated to the proof of this theorem and can be skipped without impeding the understanding of the results.

To prove that Doeblin’s condition is met, we have to evaluate the generational transitions in $\mathbf{M}_G$. In order to do this, we need to study the single mutation level first. In the mutational Markov chain $(\mathbb {N},\mathbf{M}_1)$, every state communicates with its neighbors. More precisely, it is possible to gain exactly one base pair through small insertions or duplications and to lose exactly one base pair by small deletions or large deletions. By combining these transitions, we can imagine a mutational path starting from any initial genome size to any final size, in a finite number of mutations. At the generation level, these mutational paths may exactly occur with some positive probability given by the Poisson processes. Thus all the states in $(\mathbb {N}^*,\mathbf{M}_G)$ can transit to any state in $\mathbb {N}^*$ in one step (generation).

### Concerns to Overcome

Even though all the transitions in $(\mathbb {N}^*,\mathbf{M}_G)$ are strictly positive, Doeblin’s condition is not trivially met. Because $\mathbb {N}^*$ is infinite, the probability associated to some transitions may, and will, become arbitrarily small, so there is no trivial lower bound $\varepsilon > 0$ as demanded in Doeblin’s condition. The infinite size of the matrix is the main concern here, because a number of classical theorems (such as Perron–Frobenius) do not apply. What is more, there is no absorbing state, so there is no trivial stationary distribution. The important property is that in logarithmic scale, duplications and large deletions overcome small indels and become invariant by translation (Property 1). The difficulty of the proof of Theorem 2 is that this behavior is only asymptotic (it is a good description for large genomes).

### Sketch of the Proof

We will subdivide the space of genome states in two subspaces, a finite subspace $X_\mathrm{{small}} \subset \mathbb {N}^*$ of genomes smaller than a specific size $\tilde{s}$, and an infinite subspace $X_\mathrm{{large}} \subset \mathbb {N}^*$ of genomes larger than $\tilde{s}$. We will show that a final genome size $s_f \le \tilde{s}$ can be reached in $g=2$ generations with a probability greater than a certain $\varepsilon > 0$, regardless of the starting genome size $s_0$.

- If $s_0 \in X_\mathrm{{small}}$, this condition is easily met because the subspace is finite. This will be formally stated in Lemma 1.
- If $s_0 \in X_\mathrm{{large}}$, the probability to reach $s_f$ in two generations is at least the probability to reach a state in $X_\mathrm{{small}}$ at the first generation and then to reach $s_f$ from there. For the first generation, we will show that as long as duplications are not much more frequent than deletions ($\mu _\mathrm{{dup}}< 2.59\mu _\mathrm{{ldel}}$ approximately), large genomes tend to become smaller, reach a smaller size in a finite time and stay around this smaller size. This will be formally stated in Lemma 2. We will also use Chebyshev’s inequality to show that the number of mutations in one generation is indeed sufficient to shrink below $\tilde{s}$. For the second generation, we will use again Lemma 1.

#### **Lemma 1**

Suppose we have a non-empty and finite subset of possible genome sizes $X \subset \mathbb {N}^*$. Then there is $s_f \in X$ and $\varepsilon _1 > 0$ such that $(\mathbf{M}_G)_{s_is_f} \ge \varepsilon _1$ for all $s_i \in X$.

#### *Proof*

Pick any $s_f \in X$. As $X$ is finite, the transition probabilities toward $s_f$ are bounded below by a real value $\varepsilon _1$. As just seen in the main text, every state is accessible by any other state in $(\mathbb {N}^*,\mathbf{M}_G)$ with strictly positive probability, thus $\varepsilon _1>0$.$\square$

This lemma shows that Doeblin’s condition applies trivially for $\mathbf{M}_G$ if we restrict genome size *a priori*. However, in our model, genomes may be arbitrarily large. We will show that no matter how large they are, they will reach the same finite set of states ultimately under the condition of the theorem.

#### **Lemma 2**

If $(2\log 2-1) {\mu _\mathrm{{dup}}} < {\mu _\mathrm{{ldel}}}$, there exists $\delta >0$ and a size threshold $\tilde{s}\in \mathbb {N}$ such that

- $$\forall n \ge 0, \forall s \ge \tilde{s}, \quad \mathbb {E}_{}\left[ \log (S_{n+1})|S_n = s\right] \le \mathbb {E}_{}\left[ \log (S_{n})|S_n = s\right] - \delta $$.
- $$\exists \varepsilon ' >0,$$ $$\forall n \ge 0,$$
  $$\begin{aligned} \mathrm{Pr}_{s_0}\left[ S_n \le \tilde{s}\right] \ge {\left\{ \begin{array}{ll} \varepsilon ' &{} \text {if}\; s_0 \le \tilde{s}\\ \varepsilon '\left( 1-\frac{\log s_0}{\log \tilde{s}+ n \delta }\right) &{} \text {if}\; s_0 > \tilde{s}\end{array}\right. } \end{aligned}$$

(where $\log$ is arbitrarily extended by $\log 0 = 0$)

Details of the proof for Lemma 2 are presented in Appendix 1. This proof is done by looking at the general behavior for large genomes (with size ${>}\tilde{s}$) and return times for small genomes (with size ${\le }\tilde{s}$). It involves several steps. We begin by looking at the impact of each mutation on genome size in the scale adapted to the mutations that scales most with genome size. As stated in Property 1, asymptotically, large deletions and duplications overcome local mutations and determine the spontaneous behavior. The balance between duplications and deletions decides whether large genomes will tend to grow or to shrink. If $(2 \log 2 - 1)\mu _\mathrm{{dup}}< \mu _\mathrm{{ldel}}$, the tendency is toward smaller genomes and we can find a threshold $\tilde{s}$ above which genomes shrink by at least some (relative) amount $\delta$ on average. We show that the probability for genomes to get below the $\tilde{s}$ threshold at least once progressively tends to 1, when the number $n$ of mutations increases (parenthesized part of the lower part of claim $(b)$).

In parallel, we show that a fixed fraction of genomes starting below $\tilde{s}$ remains always there. To do so, we show that starting from $\tilde{s}$ and aggregating with $\tilde{s}$ the states below $\tilde{s}$ represents a worst-case scenario in terms of genome growth. We study the time of first returns to $\tilde{s}$ in this worst-case scenario. We prove that the expected value of the first return time is finite and, using a theorem based on return times, derive the upper part of claim $(b)$. This also implies that no matter how far above $\tilde{s}$ the genome size starts, once it is reached, a fixed fraction remains there forever (explaining the presence of $\varepsilon '$ in the two parts of claim $(b)$).

As detailed below, we complete the demonstration of Theorem 2 by showing that for large genomes, the number of mutations in one generation is indeed sufficient to shrink below $\tilde{s}$ (at least asymptotically) by linking the mutation chain $(\mathbb {N},\mathbf{M}_1)$ to the generation chain $(\mathbb {N}^*,\mathbf{M}_G)$.

#### *Proof (of Theorem 2)*

We call $G_t$ the random variable that describes the state of $(\mathbb {N}^*,\mathbf{M}_G)$ at generation $t$. Let $\tilde{s}\in \mathbb {N}$ be the critical size given by Lemma 2. We subdivide the space of genome states into the finite subset $X_\mathrm{{small}}=\{ 1 \le s \le \tilde{s}\}$ and the infinite subset $X_\mathrm{{large}} = \{ s > \tilde{s}\} = \mathbb {N}\backslash X_\mathrm{{small}}$. We will show that some final genome size $s_f \le \tilde{s}$ can be reached in $g=2$ generations with a probability greater than $\varepsilon > 0$, regardless of the starting genome size $s_0$.

**Case** $s_0 \le \tilde{s}\, (s_0 \in X_\mathrm{{small}})$: The probability to reach size $s_f$ after two generations is at least the probability to reach $s_f$ and then to stay on $s_f$. We can apply Lemma 1 to $X_\mathrm{{small}}$

3 $$\begin{aligned} \exists s_f \le \tilde{s},\quad \exists \varepsilon _1 >0,\quad \forall s_0 \le \tilde{s}, \quad \mathrm{Pr}_{}\left[ G_{t+1} = s_f | G_t = s_0\right] \ge \varepsilon _1. \end{aligned}$$

This is true in particular if $s_0=s_f$.

$$\begin{aligned} \forall s_0 \le \tilde{s}, \quad \mathrm{Pr}_{}\left[ G_{t+2} = s_f | G_t = s_0\right] \ge (\varepsilon _1)^2. \end{aligned}$$

**Case** $s_0> \tilde{s}\, (s_0 \in X_\mathrm{{large}})$: The probability to reach $s_f \le \tilde{s}$ in two generations is at least the probability to reach a state in $X_\mathrm{{small}}$ at the first generation and then to reach size $s_f$ from there. We begin by considering the first step, that is, $\mathrm{Pr}_{}\left[ G_{t+1} \le \tilde{s}| G_t = s_0\right]$. This transition probability is obtained by summing the probability transitions after $n$ mutations, $(S_n)_{n \in \mathbb {N}}$, weighted by the probability that $n$ mutations occur within one generation. The number of mutations $N$ follows a Poisson distribution with parameter $\mu s_0$.

$$\begin{aligned} \mathrm{Pr}_{}\left[ G_{t+1} \le \tilde{s}| G_t = s_0\right] = \sum _{n \ge 0} \mathrm{Pr}_{s_0}\left[ S_n \le \tilde{s}\right] \frac{(\mu s_0)^n}{n!}e^{-\mu s_0}. \end{aligned}$$

According to Lemma 2,

$$\begin{aligned} \exists \varepsilon ' >0, \mathrm{Pr}_{s_0}\left[ S_n \le \tilde{s}\right] \ge \varepsilon '\left( 1-\frac{\log s_0}{\log \tilde{s}+ n \delta } \right) . \end{aligned}$$

In order for this relation to be meaningful, we look for $n^*(s_0)$ such that $\forall n \ge n^*(s_0),\, \mathrm{Pr}_{s_0}\left[ S_n \le \tilde{s}\right] \ge \varepsilon ' /2$. We find $n^*(s_0) = (2\log s_0 - \log \tilde{s}) / \delta$. This is the number of mutations that are needed to make sure that the probability of going below $\tilde{s}$ at least once is more than $1/2$. By dropping the first terms of the sum, we obtain

$$\begin{aligned} \mathrm{Pr}_{}\left[ G_{t+1} \le \tilde{s}| G_t = s_0\right] \ge \sum _{n \ge n^*(s_0)} \mathrm{Pr}_{}\left[ S_n \le \tilde{s}| S_0 = s_0 \right] \frac{(\mu s_0)^n}{n!}e^{-\mu s_0} \end{aligned}$$

thus

$$\begin{aligned} \mathrm{Pr}_{}\left[ G_{t+1} \le \tilde{s}| G_t = s_0\right] \ge \frac{\varepsilon '}{2} \mathrm{Pr}_{}\left[ N \ge (2\log s_0 - \log \tilde{s}) / \delta \right] . \end{aligned}$$

When $s_0$ goes to $+\infty$, we have $(2\log s_0 - \log \tilde{s}) / \delta \ll \mu s_0 = \mathbb {E}_{}\left[ N\right]$. What is more, $\sigma _{}\left[ N\right] = \sqrt{\mu s_0}$. This means that when $s_0$ tends to infinity, $(2\log s_0 - \log \tilde{s}) / \delta$ is below $\mathbb {E}_{}\left[ N\right]$ by a number of standard deviations that tends to infinity. The one-sided Chebyshev inequality implies that $\mathrm{Pr}_{}\left[ N \ge (2\log s_0 - \log \tilde{s}) / \delta \right]$ tends to 1. Because this nonzero limits exists and because the probability is always strictly positive, it is necessarily bounded below by some positive number. Multiplication by $\varepsilon '/2$ does not change that fact, hence

$$\begin{aligned} \exists \varepsilon _2 >0, \quad \mathrm{Pr}_{}\left[ G_{t+1} \le \tilde{s}| G_t = s_0\right] \ge \varepsilon _2. \end{aligned}$$

Once a state $s_j \in X_\mathrm{{small}}$ is reached, we apply the relation given by Lemma 1 for the second generation with the same $s_f$ as in (3)

$$\begin{aligned} \forall s_0 > \tilde{s}, \quad \mathrm{Pr}_{}\left[ G_{t+2} = s_f | G_t = s_0\right] \ge \varepsilon _2 \varepsilon _1. \end{aligned}$$

Taking $\varepsilon = \varepsilon _1 \times \min \{\varepsilon _1,\varepsilon _2\}$ gives the desired lower bound for Doeblin’s condition in two steps for $(\mathbb {N}^*,\mathbf{M}_G)$.$\square$

## Quantitative Bounds for the Distribution Using a Continuous Approximation

Theorem 2 shows that without selection, the size distribution converges toward a specific distribution $\varvec{\nu }_\infty$. From a theoretical point of view, the quantiles of this distribution give bounds that indicate where the population will asymptotically be found. However, the proof gives very little quantitative information about the location of these bounds. In order to be more precise, we need to take into account the second moments of the transition distributions. The proof of Theorem 2 relies on the fact that, asymptotically, the effect of indels become negligible compared to deletions and duplications (Property 1) already for the first-order moments. We use this remark to simplify the computations of the second moments and the bounds for the quantiles of the size distribution by considering a simplified and continuous model.

### **Definition 4**

We consider a genome with starting size $s_0 \in \mathbb {R}_+^*$ that undergoes only independent large deletions and duplications. We call $\hat{S}_{n} \in \mathbb {R}_+^*$ the genome length after $n$ mutations. The size evolution is given by $\hat{S}_{n+1} = \lambda _n \hat{S}_n$, where $\lambda _n \hookrightarrow \mathcal {U}([0,1])$ if the $n$th mutation is a deletion and $\lambda _n \hookrightarrow \mathcal {U}([1,2])$ if it is a duplication. In log scale, $\log \hat{S}_{n+1} = \log \lambda _n + \log \hat{S}_n$. We call $J_n = \log \lambda _n$. As in the general case, we assume that in one generation the number of deletions and duplications are Poisson-distributed with parameters $\mu _\mathrm{{ldel}}s_0$ and $\mu _\mathrm{{dup}}s_0$ and follow independent Poisson processes. We call $\hat{S}_f$ the genome size at the end of the generation.

### **Property 2**

Because the mutations follow independent processes, the $(J_n)_{n\in \mathbb {N}}$ are independent, identically distributed and do not depend on $\hat{S}_{n}$. What is more,

$$\begin{aligned} \mathbb {E}_{}\left[ J_n\right]&= \mathbb {E}_{}\left[ \log \lambda _n | \mathrm{del.}\right] \mathrm{Pr}_{}\left[ \mathrm{deletion}\right] + \mathbb {E}_{}\left[ \log \lambda _n | \mathrm{dup.}\right] \mathrm{Pr}_{}\left[ \mathrm{duplication}\right] \\&= -\frac{\mu _\mathrm{{ldel}}}{\mu _\mathrm{{ldel}}+\mu _\mathrm{{dup}}} + (2\log 2-1)\frac{\mu _\mathrm{{dup}}}{\mu _\mathrm{{ldel}}+\mu _\mathrm{{dup}}} \end{aligned}$$

Similarly we can obtain the second moment (thus the standard deviation)

$$\begin{aligned} \mathbb {E}_{}\left[ J_n^2\right] =\frac{2(\mu _\mathrm{{ldel}}+(1-\log 2)^2\mu _\mathrm{{dup}})}{\mu _\mathrm{{ldel}}+\mu _\mathrm{{dup}}}. \end{aligned}$$

### *Proof*

The results follow from integration by parts, namely $\mathbb {E}_{}\left[ \log \lambda _n | \mathrm{del.}\right] = \int _0^1\log x dx = -1,\, \mathbb {E}_{}\left[ \log \lambda _n | \mathrm{dup.}\right] = \int _1^2\log x dx = 2\log 2 -1$ and for the second moment $\int _0^1\log ^2 x dx = 2,\; \int _1^2\log ^2 x dx = 2(1 - \log 2)^2$.$\square$

### **Property 3**

$\mathbb {E}_{s_0}\left[ \log \hat{S}_n\right] = \log s_0 + n\mathbb {E}_{}\left[ J_n\right]$ and $\sigma _{s_0}\left[ \log \hat{S}_n\right] = \sqrt{n} \sigma _{}\left[ J_n\right]$ by the independence of the jumps. The Central Limit Theorem states that, asymptotically, $\log \hat{S}_n$ is normally distributed.

Under the continuous approximation, we have a simple jumping process that is space-homogeneous in log scale. As the two first moments are finite, it asymptotically behaves like biased diffusion because of the central limit theorem. The standard deviation increases more slowly than the mean is shifted so the expected value gives a good description of the whole distribution. The bias condition is very simple: if $\mathbb {E}_{}\left[ J_n\right] <0$, the genome shrinks on average, if $\mathbb {E}_{}\left[ J_n\right] >0$, it grows on average with every mutation. The shrinkage condition is the same as for the discrete model: genomes asymptotically shrink if and only if $\mu _\mathrm{{ldel}}> (2 \log 2 - 1) \mu _\mathrm{{dup}}$.

The main difference with the discrete case is that the relative amount by which genomes shrink is identical whatever the starting position (even for small genomes) and it can lead $\log \hat{S}_n$ to have negative values, which was not possible in the discrete space. This means that once the genome becomes small (in the sense of the proof of Theorem 2) it keeps getting smaller so there is no need to prove that it will remain small (as we did in Lemma 2). This is because there are no local mutations, which could have a strong effect on small genomes.

Thus, for large genomes, the behavior of the discrete Markov chain $(\mathbb {N},\mathbf{M}_1)$ is close to the continuous approximation and is similar to biased diffusion. When genomes become smaller, the bias may become weaker because of indels and discretization effects. On the border, the state $s = 0$ is a wall that cannot be crossed. Therefore, the discrete system is composed of a wall on one side, biased diffusion on the infinite side and an uncharacterized behavior in between. If the diffusion is biased toward the wall $(\mu _\mathrm{{ldel}}> (2 \log 2 - 1) \mu _\mathrm{{dup}})$, it is easy to imagine that the population will end up next to the wall, even though its exact final position is partly determined by small indels.

We now compute the distribution of genome size in the continuous model after one generation by weighting the $(\log \hat{S}_n)_{n\in \mathbb {N}}$ with the Poisson distribution.

### **Property 4**

By definition, for all $x \in \mathbb {R}$,

$$\begin{aligned} \mathrm{Pr}_{s_0}\left[ \log \hat{S}_f \le x\right] = \sum _{n\ge 0} \mathrm{Pr}_{s_0}\left[ \log \hat{S}_n \le x\right] \frac{((\mu _\mathrm{{ldel}}+\mu _\mathrm{{dup}})s_0)^n}{n!}e^{-(\mu _\mathrm{{ldel}}+\mu _\mathrm{{dup}})s_0}. \end{aligned}$$

The expected value is $\mathbb {E}_{s_0}\left[ \log \hat{S}_f\right] = \log s_0 + s_0 ((2\log 2 -1 )\mu _\mathrm{{dup}}- \mu _\mathrm{{ldel}})$ and the standard deviation is $\sigma _{s_0}\left[ \log \hat{S}_f\right] =\sqrt{s_0} \sqrt{2(\log 2 - 1)^2\mu _\mathrm{{dup}}+2\mu _\mathrm{{ldel}}}.$

The proof is given in Appendix 1. We introduce now a parameter $k$ that will be used to compute the fraction of the population located beyond the mean of genome size after one generation plus $k$ standard deviations. We begin by computing the latter quantity depending on $s_0$.

### **Lemma 3**

Let $k \ge 1$ and $Q_k(s_0) = \exp \left( \mathbb {E}_{s_0}\left[ \log \hat{S}_f\right] + k \sigma _{s_0}\left[ \log \hat{S}_f\right] \right)$. We call $A = \mu _\mathrm{{ldel}}- (2\log 2 -1 )\mu _\mathrm{{dup}}$ and $B=\sqrt{2(\log 2 - 1)^2\mu _\mathrm{{dup}}+2\mu _\mathrm{{ldel}}}$, so that $Q_k(s_0) = \exp (\log s_0 - As_0 + kB\sqrt{s_0})$. If $(2\log 2-1)\mu _\mathrm{{dup}}<\mu _\mathrm{{ldel}}$,

1. $Q_k(s_0)$ reaches a maximum for
  $$\begin{aligned} s_{0}^{\max ,(k)} = \frac{1}{A}+ k^2\frac{B^2}{8A^2}\left( 1+\sqrt{1+\frac{16A}{B^2}}\right) \end{aligned}$$
2. $Q_k(s_0)=s_0$ for a unique value $s_\mathrm{{fixed}}^{(k)} = k^2B^2/A^2 \ge s_0^{\max ,(k)}$.

**Fig. 2:**
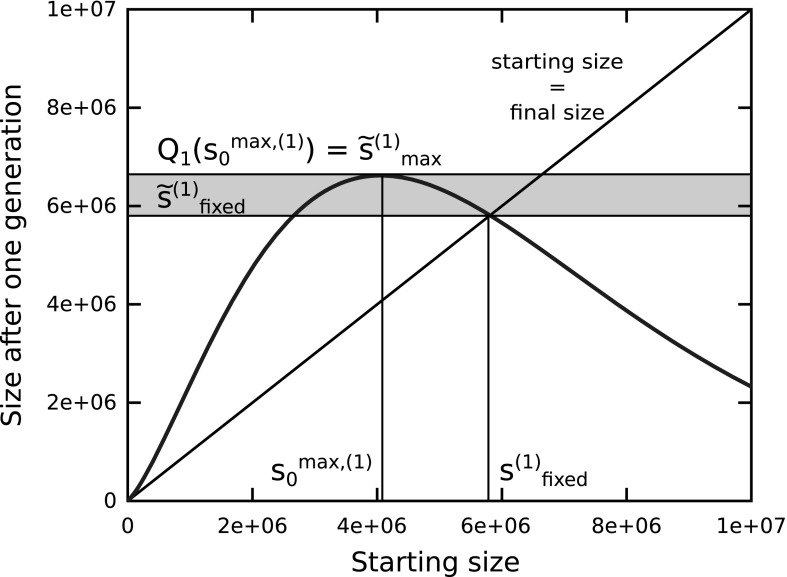
Upper bound for the median of the distribution (plot of $Q_k$ with $k=1,\,\mu _\mathrm{{dup}}=\mu _\mathrm{{ldel}}=10^{-6}$). The $x$ axis is the starting size and the $y$ axis gives an upper bound for the median after one generation. $s_\mathrm{{fixed}}^{(1)}$ is the point above which the probability that the genome shrinks is more than 0.5, $s_{0}^{\max ,(1)}$ is the starting point from which growth seems to be the most likely. A genome starting from $s_{0}^{\max ,(1)}$ may grow above $s_\mathrm{{fixed}}^{(1)}$, but it will probably shrink in the next step. The *gray area* indicates this accessible but transient set of states

The proof is straightforward and detailed in Appendix 1. The general shape of the curve and the points $s_0^{\max ,(k)}$ and $s_\mathrm{{fixed}}^{(k)}$ are depicted on Fig. 2 in the case were $k=1$ and $\mu _\mathrm{{dup}}=\mu _\mathrm{{ldel}}=10^{-6}$. By using Chebyshev’s inequality, $Q_k$ can be related to the quantiles of the distribution and we obtain the following proposition.

### **Proposition 1**

Suppose $$(2\log 2-1)\mu _\mathrm{{dup}}<\mu _\mathrm{{ldel}}$$ and $$k\ge 1$$.

1. There is a bound $\tilde{s}_{\max }^{(k)}$ such that
  $$\begin{aligned} \forall s_0 \in \mathbb {R}_+^*, \quad \mathrm{Pr}_{s_0}\left[ \hat{S}_f \le \tilde{s}_{\max }^{(k)}\right] \ge 1 - \frac{1}{1+k^2} \end{aligned}$$
  In other words, we can find a threshold $\tilde{s}_{\max }^{(k)}$ independent from $s_0$ below which an arbitrary large part of the distribution can be found after one generation of the deletion/duplication process.
2. Let
  $$\begin{aligned} \tilde{s}_\mathrm{{fixed}}^{(k)} = k^2 \frac{2(\log 2 - 1)^2\mu _\mathrm{{dup}}+2\mu _\mathrm{{ldel}}}{(\mu _\mathrm{{ldel}}- (2\log 2 -1 )\mu _\mathrm{{dup}})^2} \end{aligned}$$
  we have
  $$\begin{aligned} \forall s_0 \ge \tilde{s}_\mathrm{{fixed}}^{(k)}, \quad \mathrm{Pr}_{s_0}\left[ \hat{S}_f \le \tilde{s}_\mathrm{{fixed}}^{(k)}\right] \ge 1 - \frac{1}{1+k^2} \end{aligned}$$

### *Proof*

The proposition is a restatement of Lemma 3 by using Cantelli’s inequality (or one-sided Chebyshev inequality). It states that

$$\begin{aligned} \mathrm{Pr}_{s_0}\left[ \log \hat{S}_f \le \mathbb {E}_{s_0}\left[ \log \hat{S}_f\right] + k \sigma _{s_0}\left[ \log \hat{S}_f\right] \right] \ge 1 - \frac{1}{1+k^2} \end{aligned}$$

thus

$$\begin{aligned} \mathrm{Pr}_{s_0}\left[ \hat{S}_f \le Q_k(s_0)\right] \ge 1 - \frac{1}{1+k^2} \end{aligned}$$

The first part of the proposition follows by taking $\tilde{s}_{\max }^{(k)}=Q_k(s_{0}^{\max ,(k)})$ where $s_{0}^{\max ,(k)}$ is defined as in Lemma 3. The second part is obtained with $\tilde{s}_\mathrm{{fixed}}^{(k)}=s_\mathrm{{fixed}}^{(k)}$ in Lemma 3 and by noting that $Q'_k(s_0)<0$ for all $s_0 \ge s_\mathrm{{fixed}}^{(k)}$, so that $Q_k(s_0)\le Q_k(s_\mathrm{{fixed}}^{(k)})$.$\square$

Lemma 3 and Proposition 1 introduce two sequences of bounds that depend on a parameter $k$. $\tilde{s}_{\max }^{(k)}$ gives bounds for the quantiles of the distribution at generation $t+1$ that work for every starting genome and thus any starting distribution at time $t$. For $k=1$, the probability to get below $\tilde{s}_{\max }^{(1)}$ is at least 0.5 for every step. If the probabilities are seen as population densities, $\tilde{s}_{\max }^{(1)}$ gives an upper bound for the median of the population at any step (except maybe the starting step). Increasing $k$ increases the bound but gives even more restrictive conditions on the localization of the population at any step. For example, for $k=2$, we have $\tilde{s}_{\max }^{(2)} > \tilde{s}_{\max }^{(1)}$ but instead, we know that 80 % of the population is below $\tilde{s}_{\max }^{(2)}$ at any step.

Note that this would remain true even if the individuals were selected and then mutated. Proposition 1 says that no matter which individuals are selected (i.e., no matter the set of starting sizes $s_0$), the offspring will most likely be located below $\tilde{s}_{\max }^{(1)}$ at the next step. Figure 2 helps finding out the outcome of the selection-mutation process. To predict the impact of the mutations on size, one can interpret the $x$-axis as being the starting size and the $y$-axis as giving some likelihood about the final size. Contrary to what could be naively expected, a fitness function that would select the genomes around $s_{0}^{\max ,(1)}$ would lead to the largest genomes at the next generation, whereas a fitness function that would strongly select very large genomes would lead to much smaller genomes, as these large genomes are unable to maintain their size, even for one generation.

We also illustrate bound $\tilde{s}_\mathrm{{fixed}}^{(k)}$, which is not a bound that works for any starting distribution but whose expression is much simpler than that of $\tilde{s}_{\max }^{(k)}$. Genomes starting from $\tilde{s}_\mathrm{{fixed}}^{(1)}$ have a probability higher than 0.5 to shrink, showing that they are already strongly unstable. This shrinkage probability is far worse for genomes larger than $\tilde{s}_\mathrm{{fixed}}^{(1)}$. The analysis shows that it is possible for genomes starting around $s_{0}^{\max ,(k)}$ to increase above $\tilde{s}_\mathrm{{fixed}}^{(k)}$ but due to the definition of $\tilde{s}_\mathrm{{fixed}}^{(k)}$, this behavior can only be transient. $\tilde{s}_\mathrm{{fixed}}^{(k)}$ is a plausible upper bound for the average behavior, even when selection is applied. In the simple case where $\mu _\mathrm{{ldel}}=\mu _\mathrm{{dup}}=\mu _\mathrm{{dupdel}}$, we have

4 $$\begin{aligned} \tilde{s}_\mathrm{{fixed}}^{(k)} = \frac{k^2}{\mu _\mathrm{{dupdel}}}\frac{2(\log 2 - 1)^2+2}{(1 - (2\log 2 -1 ))^2}. \simeq 5.81 \frac{k^2}{\mu _\mathrm{{dupdel}}} \end{aligned}$$

More generally, if duplication and deletion rates are mechanically linked such that they are proportional to each other, say $\mu _\mathrm{{dup}}= \lambda \mu _\mathrm{{ldel}}$ with $\lambda < 1 / (2\log 2 -1)$, then

5 $$\begin{aligned} \tilde{s}_\mathrm{{fixed}}^{(k)} = \frac{k^2}{\mu _\mathrm{{ldel}}}\frac{2\lambda (\log 2 - 1)^2+2}{(1 - \lambda (2\log 2 -1 ))^2}. \end{aligned}$$

These relations suggest that the bound on genome size would be roughly inversely proportional to the rate of large deletions and duplications.

This analysis was done for a simplified model (continuous approximation) but it is arguably a good approximation for large genomes (genomes for which indels are negligible as in the definition of $\tilde{s}$), even in the discrete model involving all types of mutations. We expect that Proposition 1 can be obtained, with some variations, for the discrete case by showing not only that the first moment is biased (see $\delta$ in Lemma 2), but also that the standard deviation increases more slowly with the number $n$ of mutations than the first moment decreases. In this case, this would mean that, regardless of the selection applied to the genomes, we can capture an arbitrarily large part of the distribution in a finite domain at any time step.

## Generalizations and Interpretations

Theorem 2 shows that there is an asymptotic distribution $\varvec{\nu }_{\infty }$ for genome sizes in the absence of selection. The convergence of the distribution implies that an arbitrary large part of genomes is located beneath a finite size. What is more, the convergence does not depend on the rate of small insertions (possibly including transposable elements) and small deletions. For uniform distributions of duplications and large deletions, the distribution of genome sizes converges for equal rates of duplication and large deletions and even if duplications are twice as frequent and deletions. However, as mentioned in the presentation of the model, the uniform distribution for the sizes of duplications and large deletions is not necessary for the proof. In the first subsection below, we give a more general condition for the existence of a stationary distribution that encompasses a larger family of distributions. In the remainder of the section, we relate the results obtained to a more general model with selection in the case of an infinite population and their implications for a finite population.

### Extension of Theorem 2 to More General Distributions for Duplications and Deletions

We have initially hypothesized these distributions to be uniform for mathematical convenience, but all the proofs remain true under conditions similar to Property 1. All the results hold if the expected change of genome size for small indels, for duplications and large deletions converges to a constant in a specific scale given by a positive and increasing function $f$. This is the idea of invariance by translation illustrated in Fig. 1. In the general case, the existence of a stationary distribution is also determined by a condition on duplication and deletion rates (and in extreme cases indel rates) that depends only on the average size of jumps.

#### **Corollary 1**

(Generalization of Theorem 2) Suppose we have distributions of duplications, large deletions and indels, such that there exists a positive and increasing scaling function $f$ that verifies the following conditions. For $\Delta (s) = \mathbb {E}_{}\left[ f(S_{n+1}) - f(S_n) | S_n=s\right]$:

- if the (n+1)th mutation is a deletion, $\Delta (s) \underset{s\rightarrow +\infty }{\longrightarrow }\delta _\mathrm{{ldel}}$.
- if the (n+1)th mutation is a duplication,$\Delta (s) \underset{s\rightarrow +\infty }{\longrightarrow } \delta _\mathrm{{dup}}$.
- if the (n+1)th mutation is an small insertion, $\Delta (s) \underset{s\rightarrow +\infty }{\longrightarrow } \delta _\mathrm{{ins}}$.
- if the (n+1)th mutation is an small deletion, $\Delta (s) \underset{s\rightarrow +\infty }{\longrightarrow } \delta _\mathrm{{sdel}}$.

where $\delta _\mathrm{{ldel}}\le 0,\, \delta _\mathrm{{dup}}\ge 0,\, \delta _\mathrm{{ins}}\ge 0$ and $\delta _\mathrm{{sdel}}\le 0$ are constants among which at least one is nonzero.

Then the Markov chain $(\mathbb {N}^*,\mathbf{M}_G)$ has a unique asymptotic stationary probability vector $\varvec{\nu }_{\infty }$ if

6 $$\begin{aligned} \mu _\mathrm{{ldel}}\delta _\mathrm{{ldel}} + \mu _\mathrm{{dup}}\delta _\mathrm{{dup}} + \mu _\mathrm{{ins}}\delta _\mathrm{{ins}} + \mu _\mathrm{{sdel}}\delta _\mathrm{{sdel}} < 0 \end{aligned}$$

If the duplications and deletions scale more rapidly than indels, $\delta _\mathrm{{ins}}=\delta _\mathrm{{sdel}}=0$ and the condition simplifies to

$$\begin{aligned} \frac{\mu _\mathrm{{dup}}}{\mu _\mathrm{{ldel}}} < \frac{|\delta _\mathrm{{ldel}}|}{\delta _\mathrm{{dup}}} \end{aligned}$$

except for $\delta _\mathrm{{dup}}=0$, in which case there is always a stationary distribution.

The proof is the same as for Theorem 2, by replacing $(2\log 2-1)\mu _\mathrm{{dup}}-\mu _\mathrm{{ldel}}$ by the left hand-side of the new condition (inequality (6)), in particular for the definition of the global $\delta$ that incorporates the average impact of all mutations.

If the duplication and deletion processes are of multiplicative nature (but not necessarily uniform), $f = \log$ is the natural choice in the formula above, as used throughout the manuscript. If the width of the deletion and duplication distribution does not scale proportionally to $s_0$, another choice of $f$ has to be made, such that the expected change tends to a constant. In the extreme case where the average jump size already tends to a constant in normal scale $(f=id_\mathbb {N})$ for both deletions and duplications, the proof still works but the condition also incorporates the indel rates and their mean jump size. However we expect that, if the impact of indels is bounded as in our model, they will become negligible and they will not appear in the condition for realistic duplication and deletion distributions.

For example, we can consider all distributions of quasi-multiplicative nature. Corollary 1 typically applies if the losses and gains considered are relative. Roughly speaking, this happens if the distribution of gains and losses for some fraction of the genome is always the same (e.g., there is always the same probability to lose less than 5, 10% or any other fraction of the genome, no matter the initial size). In this case, the relation above applies no matter whether the distribution is exponentially decreasing, uniform, multimodal, gamma, etc. What is more, if the relative gains and losses are symmetrical (in linear scale), there will be a stationary distribution for equal duplication and deletion rates and also for duplication rates moderately higher than deletion rates (the exact relation has to be computed for each distribution).

If the second moment converges to a finite value in the scale given by $f$, as is the case for multiplicative or quasi-multiplicative distributions, the line of analysis given in Sect. 4 can be used. In other words, according to Chebyshev’s inequality, it will be possible to find bounds that will hold for every step of the generation process. As a result, as discussed below, selection will not be able to lead to infinite genome growth in these cases also. We expect that the proof can be extended for a second moment that does not converge in the scale given by $f$, but is bounded. In this case, the analysis may become more technical and the bounds given by Chebyshev’s inequality very weak, but would still imply that selection cannot lead to infinite genome growth.

### Interpretation for General Genome Structures in the Presence of Selection

We did not introduce selection in the model presented until here. In this section, we propose a more general framework for which our results hold but for which selection can be based on any feature of the genome or of the population. Let $\Omega$ be the space of all genome states corresponding to different genome architectures, e.g., all sequences of base pairs drawn from {A,C,G,T}. We define a population vector $\pi _t$ such that $\forall t \in \mathbb {N}$, $\pi _t$ is a probability measure on $\Omega$, corresponding to the density of an infinite population. We consider an arbitrary starting population $\pi _0$. For every generation, we assume that we can distinguish the selection process from the mutation process. We call $\mathrm{Sel}_t$ the selection operator. Selection may change with time. The outcome of selection has to be a probability measure whose support is expected to be identical to the support of $\pi _t$. Once selection has operated, we suppose that the population is mutated according to an operator $M$. Again, the outcome is a probability measure.

The density of the population at step $t+1$ in the space $\Omega$ of genome states is given by

7 $$\begin{aligned} \pi _{t+1} = M \circ \mathrm{Sel}_t(\pi _t) \end{aligned}$$

We suppose that genomes undergo the same mutations as those studied until here (small indels, large deletions, duplications) and other mutations that do not change genome size but the detailed architecture (e.g., point mutations, inversions, translocations). Our results apply to this model under the following condition:

*Projection condition* We suppose that there is a projection $\varphi :\Omega \rightarrow \mathbb {N}$ that is compatible with transitions induced by mutations. Here, the projection is $\mathrm{size}:\Omega \rightarrow \mathbb {N}$ that associates a genome with its size in number of base pairs. For two genomes $\omega _1,\omega _2 \in \Omega$ such that $\mathrm{size}(\omega _1)=\mathrm{size}(\omega _2)=s_0$, the probability that $\omega _1$ or $\omega _2$ end up having some size $s_f\in \mathbb {N}$ after one mutation is exactly the same, even if $\omega _1$ and $\omega _2$ have a different detailed architecture (e.g., the position or the number of genes). The transitions in the genome size space depend only on the initial genome size.

In this case, the transitions in terms of size are given by the matrix $\mathbf{M}_G$ (the additional mutations do not change genome size and thus they do not change the mutation paths in $(\mathbb {N},\mathbf{M}_1))$. To link the models formally, we define a projection $\mathbf{size}_{\varvec{\pi }}$ to obtain, from the population density $\pi _t$ in $\Omega$, the population density in the space $\mathbb {N}^*$ of genome sizes:

$$\begin{aligned} \forall s\in \mathbb {N}^*, \quad \mathbf{size}_{\varvec{\pi }}(\pi _t)(s) := \int _\Omega \mathbf{1}_{\{\omega \in \Omega , \mathrm{size}(\omega )=s\}} d\pi _t \end{aligned}$$

In matrix notation, the density of the population at step $t+1$ in the space $\mathbb {N}^*$ of genome sizes is given by

8 $$\begin{aligned} \mathbf{size}_{\varvec{\pi }}(\pi _{t+1}) = \mathbf{size}_{\varvec{\pi }}(\mathrm{Sel}_t(\pi _t)) \mathbf{M}_G\end{aligned}$$

In the absence of selection, the selection operator is the identity function, thus

9 $$\begin{aligned} \mathbf{size}_{\varvec{\pi }}(\pi _{t+1}) = \mathbf{size}_{\varvec{\pi }}(\pi _t) \mathbf{M}_G\end{aligned}$$

which is the equation studied in this article. According to Theorem 2, in the absence of selection, the marginal distribution of genome size of the population $\pi _t$ is going to converge if duplications are not more than 2.6 times more frequent than large deletions. However, we cannot show that the marginal distribution of genome size will converge in the presence of selection. Instead, we can characterize upper bounds for its quantiles.

If we consider the general model in Eq. (7) or its projection on size in Eq. (8), we see that selection occurs prior to mutations. The selection operator returns a vector $\mathrm{Sel}_t(\pi _t)$ for which the bounds found in Proposition 1 will hold. For example, by choosing $k$ for $\tilde{s}_{\max }^{(k)}$, we can say that for all $t \ge 1$, at most $100/(1+k^2) \%$ of the population contained in $\pi _{t+1}$ will have a size larger than $\tilde{s}_{\max }^{(k)}$, no matter how selection operated. Because we can find a bound that works at any generation, the spontaneous mechanism we have just described cannot be overcome by selection.

As these are upper bounds, they impose an upper limit to viable genome size but do not describe accurately where a population will be able to stabilize. This will be determined by the selection operator and the details of all mutation processes. Without further details on the selection operator, it is impossible to say whether the population will reach a stationary distribution and how far from the bounds they will evolve. Nonetheless, Fig. 2 already gives some intuition about the interactions between the selection and the mutation operators, as the size of the selected genomes has a strong impact on the outcome. As a result, selection determines how the population stabilizes with respect to these bounds and how close to the bounds individuals eventually get.

### Generalization to a Finite Population

The remarks for an infinite population hold to a lesser extent for finite-sized populations of independently mutating individuals because the results of Sects. 3 and 4 hold for a single individual from a probabilistic point of view. If we decouple the selection and the mutation steps, we can have information on the probabilities of genomes being below some bound using Proposition 1.

Basically, the idea is the same as in the infinite population case, except that the evolution of individuals is stochastic and Eq. (7) is not a good description for this kind of processes. However, if we assume that for generation $t,\, I_t$ individuals belonging to $\Omega$ survived, we can use the conclusions of Proposition 1. As explained for an infinite population, Proposition 1 allows us to choose a threshold $\tilde{s}_{\max }^{(k)}$ such that the probability that any of the $I_t$ mutating genomes goes above $\tilde{s}_{\max }^{(k)}$ is at most $1/(1+k^2)$, where $k$ can be chosen arbitrarily large. Because the individuals mutate independently, an upper bound on the number of genomes that are above $\tilde{s}_{\max }^{(k)}$ at any generation is given by a binomial distribution $\mathcal {B}(I_t,1/(1+k^2))$. The proportion of genomes supposed to be above $\tilde{s}_{\max }^{(k)}$ is the same on average for all $I_t \in \mathbb {N}: 100/(1+k^2) \%$, but the standard deviation around this proportion becomes smaller when $I_t$ increases. When $I_t$ tends to infinity, we find the same result as in the infinite case.

## Numerical Illustration and Practical Implications for the Study of Real Genomes

The theoretical results presented above may have important practical implications for the study of real genomes. To illustrate this, we present here a quantitative, numerical example of spontaneous genome size evolution, with parameter values taken from experimental data. Specifically, we simulated a “mutation accumulation” experiment on a genome made up of a single 4-Mb chromosome—which is roughly the size of the genomes of *Escherichia coli* and *Salmonella enterica*—, with spontaneous mutation rates and event size distribution derived from experimental data in both species (see details below). Mutation accumulation experiments aim at unraveling the rates and spectrum of spontaneous mutations. To do so, several lineages are propagated independently in the laboratory, starting from the same ancestor. Each lineage experiences regular, frequent single-cell population bottlenecks, so that natural selection cannot operate efficiently and evolution proceeds almost only by genetic drift. A key example of a mutation experiment with *E. coli* can be found in Kibota and Lynch (1996). Here, we mimicked an ideal mutation accumulation experiment, by simulating the evolution of genome size in 10,000 independent lines, all starting with an initial size of $4\times 10^6$ bp, during 1,000 generations, with a single-cell bottleneck at each generation. This is an “ideal” experiment in the sense that, contrary to the real laboratory experiments, we do not have to let the population grow between two bottlenecks or to pick up only the viable organisms. In the simulations, natural selection cannot act at all. No mutation will be filtered by natural selection, thereby allowing for a direct access to the spontaneous rates and spectrum of mutations.

In these simulated lineages, four types of mutations could occur: segmental duplications, large deletions, small insertions and small deletions. At each replication, the number of each type of event was drawn from a Poisson law with mean $\mu _\mathrm{{type}}s$, where $\mu _\mathrm{{type}}$ is the per-bp rate of this type of mutation and $s$ is the size of the genome before the replication. The events were performed in a random order.

The rate of large deletions $\mu _\mathrm{{ldel}}$ was set to $3.778\times 10^{-9}$ per bp, which yields, in the initial state, 0.017 deletions per genome per generation, as measured in *Salmonella enterica* (Nilsson et al. 2005). To simulate an unbiased process, we also set the segmental duplication rate $\mu _\mathrm{{dup}}$ to $3.778\times 10^{-9}$ per bp. The rates of small insertions and small deletions were both set to twice the rate of large deletions and duplications. The size of a small indel was uniformly drawn between 1 and 40 bp, regardless of the current chromosome size.

**Fig. 3:**
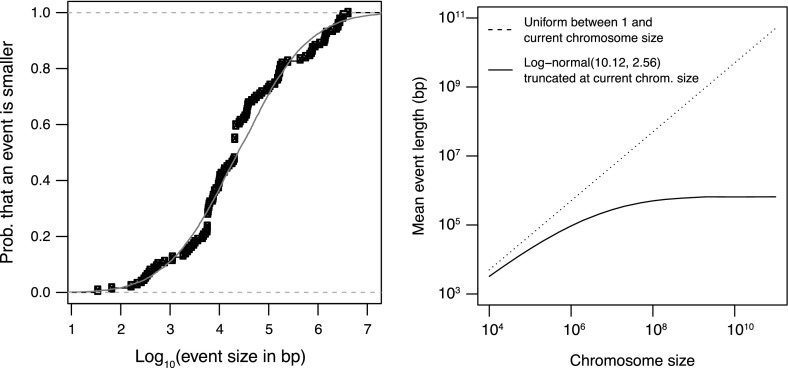
*Left* Empirical cumulative distribution function for the size (in bp) of for 107 experimentally verified rearrangements, observed in evolving cultures of *Escherichia coli* (D. Schneider, personal communication) and *Salmonella enterica* (Nilsson et al. 2005; Sun et al. 2012). The *green curve* is the distribution function of the lognormal distribution $\ln \mathcal {N}(10.1214, 2.5602)$. *Right* Expected size of a duplication or a deletion as a function of genome size, if the size is drawn from a uniform distribution between 1 and the size of the genome (*dotted line*), or if the size is drawn from the lognormal distribution $\ln \mathcal {N}(10.1214, 2.5602)$ truncated at the size of the genome (*solid line*)

For the size of duplications and large deletions, we tested two size distributions: (i) the uniform distribution between 1 and the current chromosome size, as introduced in Sect. 2, and (ii) a lognormal distribution truncated at the current chromosome size, as explained below. We obtained this lognormal distribution by fitting the size distribution of 127 rearrangements observed in evolving cultures of *Escherichia coli* (D. Schneider, personal communication) and *Salmonella enterica* (Nilsson et al. 2005; Sun et al. 2012). Only experimentally verified duplications, deletions and inversions were considered. Figure 3 (left) shows the empirical cumulative distribution function for this dataset, as well as the fitted lognormal distribution. Its mean is 10.1214 in natural logarithmic scale (4.3957 in $log_{10}$ scale) and its standard deviation is 2.5602 (1.1119 in $log_{10}$ scale). This lognormal distribution is a rather accurate representation of the events occurring on the initial 4 Mb genome. No experimental data are available, however, for the size distribution of the events occurring in mutant *E. coli* or *Salmonella* with a significantly different genome size. More generally, we do not know how the size of the spontaneous events scales with genome size in a particular species. We decided to simulate here the weakest possible scaling: instead of varying the parameters of the lognormal distribution with genome size, we used the same lognormal distribution $\ln \mathcal {N}(10.1214, 2.5602)$ for any genome size, except that this distribution was truncated at the current chromosome size. Indeed, a segmental duplication (resp. deletion) cannot duplicate (resp. delete) more than the complete chromosome. In practice, an event size was drawn from the distribution $\mathcal {N}(10.1214, 2.5602)$ with independent redraw as long as the event size exceeded the current genome size. As shown by Fig. 3 (right), this truncation induces a variation of the mean event size with the size of the genome. This variation is much weaker than the one of the uniform distribution, but it will prove important in the outcome of the simulations.

In terms of Corollary 1, for the uniform distribution, the scaling function $f$ is the logarithm. In this logarithmic scale, we know from Property 1 that $\delta _\mathrm{{ldel}} = -1,\, \delta _\mathrm{{dup}} = 2\log 2 - 1$, and $\delta _\mathrm{{ins}} = \delta _\mathrm{{sdel}}=0$. With $\mu _\mathrm{{dup}} = \mu _\mathrm{{ldel}} = 3.778\times 10^{-9}$, we obtain $\mu _\mathrm{{dup}}\delta _\mathrm{{dup}} + \mu _\mathrm{{ldel}}\delta _\mathrm{{ldel}} + \mu _\mathrm{{ins}}\delta _\mathrm{{ins}} + \mu _\mathrm{{sdel}}\delta _\mathrm{{sdel}} \simeq -2.32\times 10^{-9}$. This negative value tells us that genome size will reach a unique asymptotic stationary distribution, and hence that it will not grow infinitely.

For the truncated lognormal distribution, the scaling function $f$ is simply the identity function, thus we stay in the normal scale. For infinite genome sizes, the truncated lognormal distribution converges to the complete lognormal distribution. Hence, the event size tends to a constant for infinite genomes, and we have $\delta _\mathrm{{ldel}} = - e^{10.1214+\frac{2.5602^2}{2}} \simeq - 6.59\times 10^5,\, \delta _\mathrm{{dup}} \simeq + 6.59\times 10^5$. For the small events, we have $\delta _\mathrm{{ins}} = + 20$ and $\delta _\mathrm{{sdel}} = - 20$. With $\mu _\mathrm{{dup}} = \mu _\mathrm{{ldel}}$ and $\mu _\mathrm{{ins}} = \mu _\mathrm{{sdel}}$, we obtain $\mu _\mathrm{{dup}}\delta _\mathrm{{dup}} + \mu _\mathrm{{ldel}}\delta _\mathrm{{ldel}} + \mu _\mathrm{{ins}}\delta _\mathrm{{ins}} + \mu _\mathrm{{sdel}}\delta _\mathrm{{sdel}} = 0$. With this null value, we cannot predict the asymptotic behavior of genome size. The simulations below will show, however, that the variation of event size for “small” genome sizes (Fig. 3) suffices to induce a shrinkage.

Indeed, after 1,000 generations without selection, genome shrinkage was observed in 99 % of the lines for the uniform distribution, and in 61 % of the lines for the truncated lognormal distribution, although (i) the rates of duplications and large deletions were identical, (ii) the rates of small insertions and small deletions were identical, (iii) the event size distributions were also identical for gains and losses. Both proportions are significantly different from 0.5 ($\chi ^2$ test with 1 degree of freedom, both *p* values $< 2\times 10^{-16}$). The median DNA loss was $-3.8\times 10^6$ bp in the uniform case, and $-6.3\times 10^5$ bp in the lognormal case. In both cases, this DNA loss is statistically different from 0 (Wilcoxon signed rank test, both *p* values $< 2\times 10^{-16}$). As shown by Table 1, this shrinkage was neither due to a bias in the small indel counts nor to an excess of deletions over duplications, but to deletions being on average longer than duplications. By looking at these polymorphisms only, one might be tempted to conclude that the size distribution of the spontaneous events is different for the deletions and for the duplications, while we know here that both types of events actually had the same size distribution for any starting genome size.

**Table 1:** Median number of events and median size of observed events in a mutation accumulation line, after 1,000 generations

Uniform distribution	Large deletions	Duplications	Small deletions	Small insertions
Median number of events in a line	$4\pm 3.1$	$4\pm 5.2$	$8\pm 8.8$	$8\pm 8.7$
Median size of events in a line (in bp)	$(2.0\pm 15)\times 10^6$	$(1.1\pm 8.3)\times 10^6$	$19.8\pm 6.0$	$19.9\pm 6.1$

Lognormal distribution	Large deletions	Duplications	Small deletions	Small insertions
Median number of events in a line	$14\pm 7.1$	$14\pm 8.7$	$29\pm 14.8$	$28\pm 14.8$
Median size of events in a line (in bp)	$(1.9\pm 2.5)\times 10^5$	$(1.4\pm 1.3)\times 10^5$	$20.0\pm 2.5$	$20.0\pm 2.5$

**Fig. 4:**
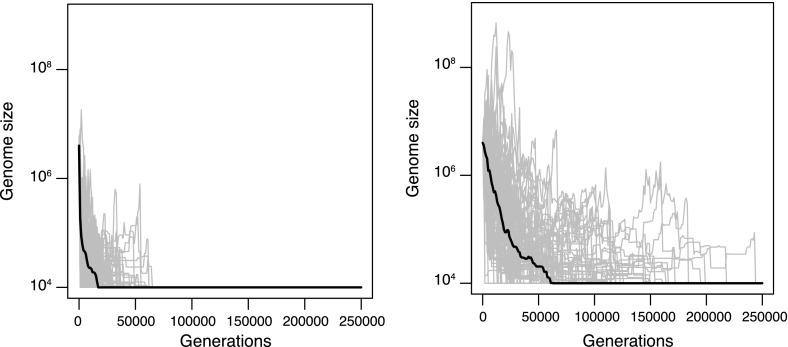
Spontaneous evolution of genome size in 100 mutation accumulation lines, under the uniform distribution (*left*) or under the truncated lognormal distribution (*right*) for the size of the rearrangements. The *thick line* indicates the median of the 100 lines. The simulations were stopped when genome size became inferior to $10^4$ (which would correspond to fewer than 10 genes in a typical bacterial genome)

To further illustrate this spontaneous mutational dynamics toward shrinkage—despite equal rates of duplication and deletion, and despite identical spontaneous size distributions for both event types—, we propagated 100 mutation accumulation lines, again without any selection, until they reached the size of $10^4$ bp, starting from $4\times 10^6$ bp as previously. As shown by Fig. 4, for all lines, this shrinkage of two orders of magnitude occurs in less than 75,000 generations for the uniform case, and in less than 250,000 generations for the truncated lognormal case.

The practical implication of this spontaneous dynamics toward shrinkage is that mutation accumulation experiments where deletions are longer than duplications should be interpreted with caution, as this pattern can be obtained with identical event size distributions. Moreover, additional simulations with higher duplication rates indicate that the median change in genome size after 1,000 generations remains negative for $\mu _\mathrm{{dup}} =1.2 \mu _\mathrm{{ldel}}$ for the truncated lognormal distribution, or for $\mu _\mathrm{{dup}} = 2 \mu _\mathrm{{ldel}}$ for the uniform distribution (in agreement with Theorem 2). Thus, a net decrease in genome size in a mutation accumulation experiment neither implies that the deletion rate is higher than the duplication rate, nor that spontaneous deletions tend to be longer than spontaneous duplications for a given starting point. It might just as well come from a variation of event count and event size with chromosome size.

## Discussion

In this section, we discuss the relevance of our two main results: (i) the condition for the existence of a stationary distribution for genome size, and (ii) the upper bounds for the quantiles of the genome size distribution at any time step. Finally, we investigate the link between our results and current theories on genome size evolution.

### On the condition for the existence of a stationary distribution for genome size

The model and the results presented here allow to determine the global spontaneous behavior of genome size in a variety of conditions. It allows for a large family of distributions for indels, duplications and deletions. Conveniently, the condition for the existence of an asymptotic distribution only depends on the first moment of these distributions in a scale adapted to the distribution that scales most with genome size (usually large deletions and/or duplications). The most difficult part may be finding the scaling function, but once it is found, the global dynamics is dictated by a very simple condition on the mutation rates, given in Corollary 1. Usually, the condition will only involve the ratio of duplication over large deletion rates. When the condition is met and no selection is applied, the genomes converge toward an asymptotic stationary distribution. From a biological point of view, this means that genomes do not grow indefinitely. As our proof highlights, there is a threshold above which they will undergo systematic shrinkage.

Finding the appropriate scale might be one of the most important challenges but can also be very simple for some families of distributions. For example, if the duplication and deletion processes are of quasi-multiplicative nature, this condition is obtained in logarithmic scale, as illustrated throughout the paper and in Fig. 1. Biologically speaking, the strength of this scaling is that it breaks apparent symmetries. In the case illustrated in Fig. 1, it might look as if the duplications and deletions are symmetrical because *for every starting position*, losses and gains compensate each other. Naively, one might conclude that the process is unbiased. However, from the genome’s point of view as a walker along a Markov chain, symmetry means reversibility of jumps: do the losses and gains that I undergo *when I move* compensate each other? If there is a scaling in which the average size of jumps does not depend (asymptotically) on the starting position, we can answer the question. In the rescaling shown in Fig. 1, it is clear that the process that we thought unbiased at first, is in fact biased toward losses. Indeed, after a loss is undergone, the average size of rearrangement diminishes with genome size, such that the average loss will always be larger than the next average gain.

As shown in Sect. 6, even if we choose a function that does not scale asymptotically (average gains and losses due to rearrangements tend to a constant for large genomes in normal scale), a scaling in gains and losses for small genomes will still induce a bias toward losses. The process based on the truncated lognormal distributions for rearrangements that we illustrated might not converge in theory for equal duplication and deletion rates (Corollary 1 does not apply) but, in practice, the bulk of genomes will still undergo shrinkage because average losses are larger than average gains, notwithstanding the symmetry of the rearrangement distributions.

From a mathematical point of view, our model displays similarities with models for the so-called mini- and micro satellite loci, where a short sequence of DNA is highly repeated (Charlesworth et al. 1994). Mathematical models were designed for the dynamics of the number of repeats in microsatellites, incorporating additive mechanisms similar to indels in our model and/or multiplicative mechanisms due to recombination. In models incorporating only additive effects, the dynamics of the number of elements is relatively simple as it reflects the difference between average gains and losses (Krüger and Vogel 1975; Walsh 1987; Moody 1988; Basten and Moody 1991; Caliebe et al. 2010). However, this implies that selection is necessary to prevent the number of repeats from going down to one or from going up to infinity. When multiplicative effects are introduced, the dynamics become less trivial, as noted in the present study. Distributions of the number of elements can converge around a finite number of elements in situations where average gains seemed to be higher than average losses (Stephan 1987; Falush and Iwasa 1999).

Adding additive effects (such as indels) to a multiplicative model does not change the existence of the stationary distribution but changes some important features. Corollary 1 implies that when multiplicative and additive processes are at work, we can have non-trivial stationary distributions without the need for selection. This is particularly true in non-intuitive cases, when the apparent bias is toward gains, but the rescaled analysis predicts average loss. In those cases, the mode of the distribution cannot be trivially predicted to lie at the origin or to diverge. For example, Falush and Iwasa (1999) predicted a non-trivial stationary distribution for the number of repeats, where most individuals own more than one or two copies. Compared to the microsatellite models, the strength of our result is that it can be used to predict the exact threshold condition with simple calculations, without having to make any approximation. Moreover, in most models where the size of the mutation scales with the number of elements, each mutation type is allowed to occur at most once per reproduction, which may be appropriate for the study of microsatellites, but unrealistic for large genomes.

The interplay between additive and multiplicative processes might also be underlined in the study of genome reduction. Mira et al. (2001) have argued that indel biases and losses through large deletions are good candidates to explain the reductive genome evolution undergone by some bacterial species. Several models and papers have used this general idea but have focused only on biases in the small indel patterns (Petrov 2000; Leushkin et al. 2013). Our numerical examples (Sect. 6) show that such a bias is not necessary for observing genome shrinkage, as it suffices that there be a positive scaling in the size of rearrangements. A symmetrical distribution of gains and losses, or even a slight bias toward gains will result in genome size tending to decrease with time (Fig. 4). The “rearrangement bias” (due to the scaling of rearrangements) and the indel bias are two different biases that might be complementary. According to our model and simulations, the rearrangement bias would set an upper size to genome size but would not necessarily lead to the convergence toward a particular value below this bound (notably in the presence of selection), while the indel bias could not lead to infinite growth but would strongly affect the convergence of genome size within stable genomes (see discussion and figure in Sect. 7.3).

### On the Upper Bounds for the Quantiles of the Genome Size Distribution

In the model presented here, we took into account the possibility of several mutations in one generation. This does not change the condition for a stationary distribution given by the first-order dynamics, but highlights a fragility of large genomes, which we quantified by calculating upper bounds for the quantiles of the genome size distribution after one *generation*. Contrary to the mutational Markov chain, the generational Markov chain shows that there is a non-negligible probability that the genomes located above a given threshold, no matter how large they may be, collapse. Strikingly, as already hinted in Lemma 2 and the proof of Theorem 2, once the threshold is crossed, the probability of collapsing even increases with genome size, because the average loss due to the increasing number of mutations grows more rapidly than the genome size.

In order to quantify this phenomenon further, including the second moment gave a more precise picture. In the case of multiplicative processes, the standard deviation is small compared to the average shifts, such that the average behavior identified in Sect. 3 gives in fact a good picture of the fate of large genomes. This is illustrated in Fig. 2, where it appears that after one generation, the genomes which manage to maintain or increase their size with probability higher than 0.5 are restricted to a finite domain.

The existence of these bounds has two main implications. First, they are not bounds on the stationary distribution but on the whole process. This means that even if a selective force is applied, they are verified for every surviving individual. In other words, selection cannot help overcome these bounds, even if the selection operator favors the largest genomes. On the contrary, as depicted on Fig. 2, very large genomes that may be selected are going to be the least robust, as they become even smaller than the rest of the population and their genome is going to undergo major shuffling. This result is in contrast with other models for evolution with selection on a unbounded fitness space, which showed that the growth speed of the first moment of the fitness distribution converges to a positive constant, even when a density-dependent cut-off prevented the fittest individuals to replicate (Tsimring et al. 1996; Brunet and Derrida 1997). We do not need here such a cut-off to prevent infinite genome growth, even if selection would favor the largest genomes. To analyze more rigorously our model in the presence of selection, a possibility would be to consider a Markov process in the space of measures on $X$, as in the evolutionary models proposed by Fleming and Viot (1979), Champagnat et al. (2006).

Second, the individuals who have important probabilities to maintain their size are those which undergo rare rearrangements. In this sense, our model *predicts* as a result what is *assumed* by numerous models, namely that the majority of a robust population will undergo at most one rearrangement (or even one mutation if the rates are similar) per generation. Allowing for several mutations in one generation highlights an indirect pressure that limits genome growth but that is not necessarily likely to be observed in a sample of the population.

We predicted at the end of Sect. 4 an inverse relationship between total genome size and mutation rate, in the case where the deletion and duplication rate are proportional (Eq. 5). This relationship is strikingly similar to Drake’s experimental data for microorganisms (Drake 1991), where genome size seems to be inversely proportional to the “global mutation rate”. However, in Drake’s study, most spontaneous events used to determine the global rate are of local nature (indels, small rearrangements and point mutations), while in our study, the critical rates are the rates of chromosomal rearrangements.

**Fig. 5:**
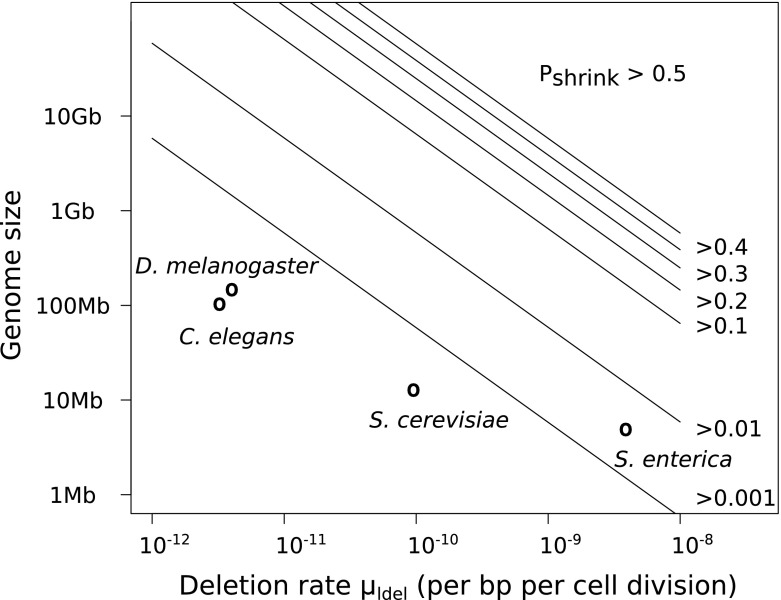
Comparison of the bounds on genome size derived in Sect. 4, Eq. (4) with the genome size for four organisms. Spontaneous deletion rates were computed per base pair and per cell division from experimental data on mutation accumulations for the bacterium *Salmonella enterica* (Nilsson et al. 2005), the budding yeast *Saccharomyces cerevisiae* (Lynch et al. 2008), the worm *Caenorhabditis elegans* (Lipinski et al. 2011) and the fruit fly *Drosophila melanogaster* (Schrider et al. 2013). The value next to each line is the lower bound for the probability that a genome located along this line will shrink at the next step in our model for equal duplication and deletion rates (Eq. 4, Sect. 4)

Thus, to better assess the relevance of the relationship we predicted, we need to know more about the actual rates of duplications and deletions. Conflicting data sets exist on the link between allelic recombination rates and genome size (see Awadalla (2003) and Ross-Ibarra (2007) for example), but deletions and duplications are non-allelic recombination events, and the rate of non-allelic recombination is not necessarily directly proportional to the rate of allelic recombination. Indeed, allelic recombination results from homologous recombination only, while non-allelic recombination can result from other mechanisms. In human, Turner et al. (2008) have measured the spontaneous rates of non-allelic recombination events leading to deletions and duplications, but only at four recombination hotspots. So far, genome-wide measurements of duplication and deletion rates have been obtained by mutation accumulation experiments for a few species only. Figure 5 shows the available data points, as well as the lower bounds for genome shrinkage computed in Sect. 4, Eq. (4). We observe that genomes stabilize in zones were rearrangements are rare and the probability to shrink is very low, far below the bounds were genomes start to become unstable. The precise dynamics of genomes in the presence of selection depends on the selection operator, so our model cannot precisely predict how far below the bounds real organisms are going to be.

The existence of our bounds relies on rates of chromosomal rearrangements that are expressed as rates per base pair to determine how often they occur in one generation. Because of the Poisson law, the number of expected rearrangements increases linearly with genome size. As genome length may change after every rearrangement, this hypothesis may seem strong. For example, even if a large part of the genome was lost after a large deletion, the number of rearrangements remaining is given by the Poisson law based on the initial genome length, so the genome will continue to mutate several times if the initial size was large. It would be interesting to study the case where the rates of the Poisson process take into account the current genome size (the process would not be Poisson anymore) in order to reevaluate the number of mutations remaining. Preliminary results indicate that the results would still hold but that the curve giving the median of the size after replication as a function of the starting size would increase monotonically to a finite limit (instead of decreasing for large genomes as on Fig. 2). Alternatively, it could be interesting to estimate the number of rearrangements based on a mechanical model.

From a biochemical point of view, the rearrangement rate should not be expressed as a per base rate, but depending on elements that drive rearrangements (in which case we could look for a projection $\varphi$ on these elements instead of the mapping on size). To study the relevance of our analysis, a deeper biochemical understanding of the scaling of rearrangement rate and size with genome size is needed. Some rearrangements are known to be driven by specific sequences, such as transposable elements, insertion sequences or tandemly repeated sequences. Biological data indicate that the number of transposable elements scales with genome size (Oliver et al. 2007), and in *Arabidopsis* the reduction of genome size could be linked to the capacity of one family of transposable elements to mediate rearrangements (Devos et al. 2002).

The impossibility of long-term accurate replication for large genomes reminds of Eigen’s error threshold (Eigen 1971; Eigen et al. 1988). Eigen noted that the mutation rate puts a limit on the size of a replicating polymer. If a molecule exceeds this critical size, the number of mutations per replication is so high that the information is destroyed in subsequent generations of the molecule. This model is often considered relevant for viruses, which have small genome sizes and high point mutation rates (Eigen and Schuster 1979; Nowak 1992; Wilke 2003). Although the original formulation of Eigen’s model was rather general, the error threshold prediction was derived for the special case where all sequences have the same fixed length and undergo only point mutations (Eigen 1971; Eigen et al. 1988). Subsequent studies have relaxed other assumptions such as the infinite population size (Nowak and Schuster 1989) or the homogeneity of the mutation rate along the sequence (Barbosa et al. 2012), but in all cases the mutations considered remained the local ones, although the importance of duplications and deletions was discussed in Eigen et al. (1988). Other models were designed to tackle related questions such as the existence of an error threshold limiting the total number of essential genes (Zeldovich et al. 2007) or the extension to a cost including transcription or translation errors (Bird 1995; Pál and Hurst 2000).

Our study shows that the nature of the mutations included in the model is important when studying the evolution of the genome structure as a whole, possibly including coding and noncoding DNA. If only local mutations are considered, then the maximum size rule applies only to the coding part of the genome (Eigen et al. 1988). We have shown here that if rearrangements with global effects are considered as well, then the noncoding part of the genome is also bounded, because noncoding DNA is mutagenic for the surrounding genes, as it provides breakpoints for large duplications and deletions. This phenomenon was observed in an individual-based model of genome evolution where genomes were explicitly represented as variable-length binary strings, and where an artificial chemistry was defined to compute the fitnesses (Knibbe et al. 2007). Knibbe’s model fits into our framework and corresponds to the finite-sized population case discussed in Sect. 5. By including rearrangements in Eigen’s original model, we predict the existence of a generalized error-threshold applicable to the whole genome.

### On the Link with Current Hypotheses in Molecular Evolution

Genome growth is generally explained by the self-replicating activity of transposable elements and by the neo- or sub-functionalization of gene duplicates (Lynch and Conery 2003), while the mechanisms pushing toward genome reduction are less clear. Our results reveal that the sole dynamics of large duplications and large deletions implies a subtle bias toward genome shrinkage. In the model, genomes tend to shrink even if the duplication and deletion rates are equal because of the multiplicative nature of these events. Thus, the chromosomal instability of large genomes presented here is one of the pressures that can oppose genome growth. However, it is not the only pressure acting on genome size. As detailed below, other models hypothesize that total genome size has a direct fitness cost, that transposable element insertions are deleterious or that indels play a prominent role.

Some theories suggest that genome size could be directly selected. Long genomes could be longer to replicate and have a higher metabolic cost than smaller ones and thus be counter-selected (Maniloff 1996; Poole et al. 2003). This hypothesis is now largely rejected, for example Mira et al. (2001) found no correlation between doubling time and genome size across diverse bacterial taxa, probably because replication can start anew on a genome already engaged in replication, and because there is much more material than DNA that has to be copied and shared during division. Alternatively, the size of the nucleus (and, directly or indirectly, of the cell) could be linked to the bulk DNA content. In this hypothesis, an optimal cell size is selected for physiological reasons and the large variations of non-coding DNA are interpreted as a way to control precisely the size of the nucleus (Cavalier-Smith 1978, 1985; Gregory 2001; DeLong et al. 2010). Our model suggests that if selection favors a specific size, convergence of the population is possible only if this optimal size is in the zone where chromosomal rearrangements are rare. In other words, a larger DNA content is possible only if molecular mechanisms evolve that increase the stability of chromosomes by reducing the frequency of duplications and large deletions.

Other theories focus more specifically on the gene repertoire and events that can affect it. On the one hand, transposition of transposable elements can lead to genome growth but their insertions can be deleterious either because they disrupt genes or because they promote ectopic recombination and thus, possibly, large deletions. Because of these deleterious effects, selection would naturally eliminate transposable elements of the genome. However, population genetics predicts that, in populations with smaller effective sizes, selection will be less likely to eliminate them and the genome will be larger . Accumulation of selfish elements would thus reflect small population sizes (see Lynch and Conery (2003) for further details).

On the other hand, some explanations invoke a higher spontaneous rate of small deletions compared to small insertions, with a bias strong enough to prevent the genome from growing (Petrov 2000). This bias is due to more frequent or larger small deletions compared to small insertions and it is usually detected because it leads to an erosion of non-coding sequences (Ophir and Graur 1997; Mira et al. 2001; Kuo and Ochman 2009; Leushkin et al. 2013). In this case, smaller population sizes can lead to *smaller* genomes. Indeed, because of random genetic drift, some non-essential genes may be inactivated and, subsequently, the resulting pseudogenes may be eroded by accumulation of small deletions (Mira et al. 2001). More generally, the evolution of genome size in small populations will be more influenced by mutational biases.

**Fig. 6:**
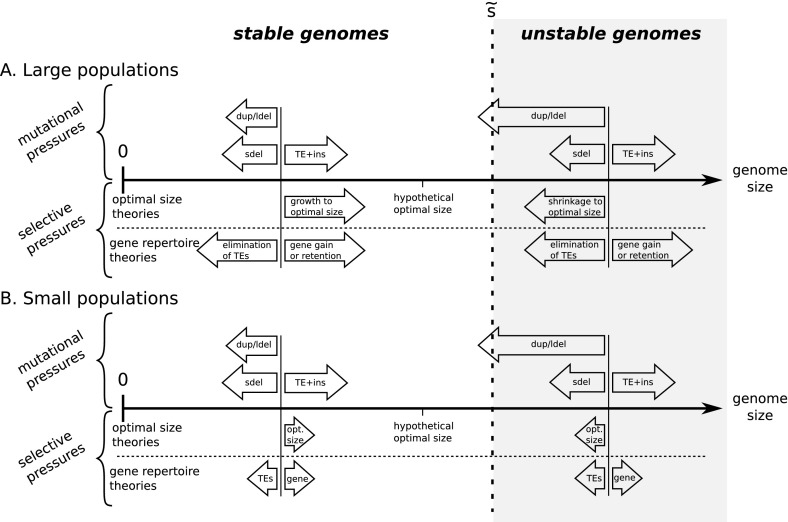
**Sketch of mutational pressures and selective pressures according to the theories in the literature**. *Arrows* indicate the schematic strength of each pressure. The mutational *arrows* indicate the average size of each type of spontaneous mutation: shrinkage pressure due to large duplications and large deletions, shrinkage pressure due to small deletions and growth pressure due to small insertions and transposable elements (TEs). Whereas the average impact of a small deletion, a small insertion or a TE insertion does not depend on genome size, the average loss due to duplication and large deletion events scales with genome size (see Sect. 2.2). Our model predicts that this mutational bias overcomes other mutational and selective pressures for genomes larger than a certain threshold $\tilde{s}$ (as defined in Lemma 2). Below the threshold, other pressures will play a more significant role and the genome size at equilibrium should depend on the precise intensity of each mutational pressure for the species considered, and on the effective population size. **a** In large populations, the selective pressures identified in the literature (see main text for details) can be strong enough to overcome these mutational pressures except for very large genomes which cannot be maintained because of strong chromosomal instabilities according to Proposition 1. **b** In small populations, the mutational pressures do not change but, according to population genetics, the selective pressures are less efficient. Because of genetic drift, more spontaneous deleterious events are fixed in the population. The dynamics of the population should therefore be more influenced by spontaneous events, e.g., transposition of transposable elements, biases in small indels or increased ectopic recombination. Still, even if these pressures drive the genome toward expansion, the bias identified in this article will quickly grow and overcome them, keeping the genome under a finite bound.

We predict that above some size threshold, the mutation bias due to ectopic recombination identified here will be stronger than any other mutational bias and even stronger than selective pressures: it will bring the genome back under the stability threshold. For stable chromosomes (which undergo fewer and smaller rearrangements), the dynamics of genome size will be influenced by selection (if the population is large), transposition of transposable elements, the tendency to genome shrinkage due to ectopic recombination as identified here and the biases in the small indels. Therefore, the equilibrium genome size depends on the strength of each of these forces, which will depend on the species considered (Fig. 6).

Indeed, large variations exist within closely related species (Thomas 1971; Betrán and Long 2002; Tenaillon et al. 2011), indicating that there might not be one explanation valid for all species. While a spontaneous bias toward small deletions compared to small insertions seems to be widespread among bacteria (Kuo and Ochman 2009), some species exhibit an opposite bias toward insertions (Denver et al. 2004). Similarly, transposable elements are rare in some species (supposedly in bacteria for example) and their transposition rate may be controlled by the host, so that the interplay between small indels, large deletions and transposition has to be evaluated for each species.

To conclude, several evolutionary pressures act together on real genomes. The advantage of modeling studies such as the present one is that one can isolate and investigate a given pressure such as, here, the spontaneous formation of deletions and duplications through ectopic recombination.

## Appendix 1: Existence of an Asymptotic Distribution: Detailed Proof of Lemma 2

### *Remark 1*

In the whole section, we will consider $\log$ as a function defined on $\mathbb {N}$ with $\log 0 = 0$ and the usual values for $n\ge 1$, such that $\log$ is a positive and increasing function on $\mathbb {N}$.

The section is dedicated to the proof of Lemma 2. Before the actual proof, we will list properties of the Markov chain $(\mathbb {N},\mathbf{M}_1)$. We will focus on the fate of small genomes on the one hand and of large genomes on the other hand.

### Properties of Large Genomes

We begin with the properties of large genomes. We start with the proof of Property 1, in a slightly more detailed version. This property, introduced in Sect. 2, states that, asymptotically, duplications and large deletions overcome small indels and their average impact tends to a constant in logarithmic scale.

#### **Property 5**

(Detailed version of Property 1) Let $\Delta (s)= \mathbb {E}_{}\left[ \log (S_{n+1})|S_n = s\right] -\mathbb {E}_{}\left[ \log (S_{n})|S_n = s\right]$.

- - if the (n+1)th mutation is a deletion
    $$\begin{aligned} \forall s\ge 3, \quad \Delta (s) = -1 + \frac{1}{s} \left( \sum \limits _{k=2}^{s-1}\log k - \int _{0}^{s}\log x \mathrm{{d}}x \right) \underset{s\rightarrow +\infty }{\longrightarrow } -1 \end{aligned}$$
  - if the (n+1)th mutation is a duplication
    $$\begin{aligned}&\forall s\ge 3, \quad \Delta (s) = 2\log 2-1 \\&\quad + \frac{1}{s} \left( \sum \limits _{k=s+1}^{2s}\log k - \int _{s}^{2s}\log x \mathrm{{d}}x \right) \underset{s\rightarrow +\infty }{\longrightarrow } 2\log 2-1 \end{aligned}$$
  - if the (n+1)th mutation is a small deletion resp. a small insertion
    $$\begin{aligned} \Delta (s) \underset{s\rightarrow +\infty }{=} O\left( \frac{1}{s} \right) \underset{s\rightarrow +\infty }{\longrightarrow } 0 \end{aligned}$$
- In the general case
  $$\begin{aligned} \Delta (s) = \frac{(2\log 2-1) \mu _\mathrm{{dup}}- \mu _\mathrm{{ldel}}}{\mu } + \xi (s) \end{aligned}$$
  where $$\lim \limits _{s \rightarrow +\infty } \xi (s) = 0$$.

#### *Proof*

If the (n+1)th mutation is a deletion,

$$\begin{aligned}&\mathbb {E}_{}\left[ \log (S_{n+1})|S_n = s\right] = \sum \limits _{k \ge 0}\log (k) \mathrm{Pr}_{}\left[ S_{n+1} = k|S_n=s\right] = \frac{1}{s}\sum \limits _{k = 2}^{s-1}\log k\\&\quad = \frac{1}{s}\left( s(\log s-1) + \sum \limits _{k = 2}^{s-1}\log k - \int _{0}^{s}\log x \mathrm{{d}}x \right) \\&\quad = \log s - 1 + \frac{1}{s}\left( \sum \limits _{k = 2}^{s-1}\log k - \int _{0}^{s}\log x \mathrm{{d}}x \right) \end{aligned}$$

The result easily follows as $\mathbb {E}_{}\left[ \log (S_{n})|S_n = s\right] =\log s$. The demonstration for duplications is similar and trivial for the others mutations given the definition of transitions.

Result (b) comes from the law of total expectation conditioned on every type of mutation. The limits and the fact that $\xi (s)$ vanishes when $s \rightarrow \infty$ come from simple sum-integral comparisons and Taylor expansions. For deletions, log being an increasing function:

$$\begin{aligned} \forall k \ge 2, \quad \log k \le \int _k^{k+1}\log (x)\mathrm{{d}}x \le \log (k+1) \end{aligned}$$

Summing over $k \in \{2,\ldots ,s-1\}$ for some $s \ge 3$

$$\begin{aligned} \sum \limits _{k=2}^{s-1}\log k \le \int _2^{s}\log (x)\mathrm{{d}}x \le \sum \limits _{k=2}^{s-1} \log (k+1) = \sum \limits _{k=2}^{s-1}\log k - \log 2 + \log s \end{aligned}$$

reorganizing each side separately and multiplying by $1/s$

$$\begin{aligned} \frac{-\log s + \log 2}{s}\le \frac{1}{s}\left( \sum \limits _{k=2}^{s-1}\log k - \int _2^{s}\log (x)\mathrm{{d}}x\right) \le 0 \end{aligned}$$

which vanishes when $s\rightarrow +\infty$. The missing integral term $(\int _0^2\log (x)\mathrm{{d}}x)$ is constant and also vanishes when divided by $s$. Changing the summation subsets yields the same result for duplications.

For small deletions, let $f_s(k)=\mathrm{Pr}_{}\left[ S_{n+1} = k|S_n=s\right]$ knowing that a small deletion happened. For $s \ge l_\mathrm{{sdel}}$

$$\begin{aligned} \Delta (s)&= \sum \limits _{k=-l_\mathrm{{sdel}}}^{-1}f_{s}(s+k)(\log (s+k) - \log s) = \sum \limits _{k=-l_\mathrm{{sdel}}}^{-1}f_{s}(s+k)\log ( 1 + k/s ) \\&\underset{s\rightarrow +\infty }{=} \sum \limits _{k=-l_\mathrm{{sdel}}}^{-1} f_{s}(s+k) O\left( \frac{1}{s}\right) = O\left( \frac{1}{s}\right) \end{aligned}$$

The result is symmetrical for small insertions.

Unconditioning leads to multiplying these terms by constants, $\lim _{s\rightarrow +\infty }\xi (s)=0$.$\square$

Asymptotically, the average bias is determined by $(2\log 2-1) \mu _\mathrm{{dup}}- \mu _\mathrm{{ldel}}$. We are interested in the case where genome tend to shrink, so we sum up the previous property in a new property restricted to our scope.

#### **Property 6**

If $(2\log 2-1) \mu _\mathrm{{dup}}- \mu _\mathrm{{ldel}}<0$, we call $\delta = |(2\log 2-1) \mu _\mathrm{{dup}}- \mu _\mathrm{{ldel}}|/(2\mu )>0$. There is a threshold size $\tilde{s}\in \mathbb {N}$ such that

$$\begin{aligned} \forall n \ge 0, \forall s \ge \tilde{s}, \quad \mathbb {E}_{}\left[ \log (S_{n+1})|S_n = s\right] \le \mathbb {E}_{}\left[ \log (S_{n})|S_n = s\right] - \delta \end{aligned}$$

This is only a rephrasing of Property 5(b) by using the definition of the limit.

#### *Remark 2*

This is a strong property as it applies to any distribution at step $n$ whose support is above $\tilde{s}$. In other words, we can change the conditioning $S_n=s$ to any of these distributions, the property still holds because of the theorem of total expectation.

### Properties of Small Genomes

We begin by showing that, statistically, a genome with a smaller size will give smaller genomes after one mutation.

#### **Property 7**

$$\forall s_1, s_2 \in \mathbb {N}$$, $$s_1 \le s_2 \Rightarrow \mathrm{Pr}_{}\left[ S_{n+1} \le k|S_n=s_1\right] \ge \mathrm{Pr}_{}\left[ S_{n+1} \le k|S_n {=} s_2\right] $$

#### *Proof*

Let $F_{s}(k) = \mathrm{Pr}_{}\left[ S_{n+1} \le k|S_n=s\right]$. We use $\mathbf{1}_{\{s,\ldots \}}:= \mathbf{1}_{\{k \in \mathbb {N}, k\ge s\}}$.

We condition on the type of the $(n+1)$th mutation. If the mutation is a deletion, $F_{0}=\mathbf{1}_{\mathbb {N}}$ and if $s>0$, $F_{s}(k) = (k+1)\mathbf{1}_{\{0, \ldots , s-1\}}(k)/s + \mathbf{1}_{\{s, \ldots \}}(k)$. For a duplication, $F_{0}=\mathbf{1}_{\mathbb {N}}$, if $s>0,\, F_{s}(k) = (k-s)\mathbf{1}_{\{s+1,\ldots ,2s\}}(k)/s + \mathbf{1}_{\{2s+1,\ldots \}}(k)$. Because the transitions for indels are invariant by translation, we have $F_{s_1}(k)=F_{s_2}(k + s_2-s_1) \ge F_{s_2}(k)$ as $s_2-s_1 \ge 0$.

Let $s_1 \le s_2 \in \mathbb {N}$. For every type of mutation, $\forall k \in \mathbb {N}$, $F_{s_1}(k) \ge F_{s_2}(k)$. Unconditioning by multiplying by the probability of each mutation does not change this fact as it is a weighted average.$\square$

This property is useful in the following when we show that small genomes tend to remain small. Indeed, if we can show that some genome of size $s_\mathrm{{min}}$ tends to stay small, we expect that it is also true for any genome of smaller size (but another property could be used, see Remark 4 below). We give this a formal meaning by introducing the following definition.

#### **Definition 5**

Let $s_\mathrm{{min}} > 0$. We define a subprocess $S^\rhd _n$ on $(\mathbb {N}^\rhd ,\mathbf{M}_1^\rhd )$ as follows. We keep only the states larger or equal to $s_\mathrm{{min}}$ in $\mathbb {N}$, so $\mathbb {N}^\rhd =\{s \in \mathbb {N}: s \ge s_\mathrm{{min}}\} \subset \mathbb {N}$. The transitions in $\mathbf{M}_1^\rhd$ are the same as in $\mathbf{M}_1$, except those which go below $s_\mathrm{{min}}$ which are rewired to $s_\mathrm{{min}}$. Formally,

$$\begin{aligned} \forall i&\ge s_\mathrm{{min}}, \forall j > s_\mathrm{{min}}, \quad \mathrm{Pr}_{}\left[ S^\rhd _{n+1} = j | S^\rhd _n=i \right] \\&= (\mathbf{M}_1^\rhd )_{ij} := (\mathbf{M}_1)_{ij} = \mathrm{Pr}_{}\left[ S_{n+1} = j | S_n=i \right] \end{aligned}$$

and

$$\begin{aligned} \forall i \ge s_\mathrm{{min}}, \quad (\mathbf{M}_1^\rhd )_{is_\mathrm{{min}}} := \sum _{0 \le k \le s_\mathrm{{min}}} (\mathbf{M}_1)_{ik} = \mathrm{Pr}_{}\left[ S_{n+1} \le s_\mathrm{{min}} | S_n=i \right] \end{aligned}$$

We begin by some simple properties of the Markov chain $(\mathbb {N}^\rhd ,\mathbf{M}_1^\rhd )$.

#### **Property 8**

If $\max \{\mu _\mathrm{{sdel}},\mu _\mathrm{{ldel}}\}>0$ and $\max \{\mu _\mathrm{{ins}},\mu _\mathrm{{dup}}\}>0,\, (\mathbb {N}^\rhd ,\mathbf{M}_1^\rhd )$ is irreducible and aperiodic.

#### *Proof*

Irreducibility means that it is possible to get from any state $s_1 \in \mathbb {N}^\rhd$ to any other state $s_2\in \mathbb {N}^\rhd$ in an arbitrary number of steps. As the mutations span the neighboring states (on both sides if the condition of the property is satisfied), this is easy to verify. The periodicity of a given state $s\in \mathbb {N}^\rhd$ is given by $\gcd \{n \in \mathbb {N}: \mathrm{Pr}_{s}\left[ S^\rhd _n=s\right] >0\}$. As the chain is irreducible, it is aperiodic if there is a state $s\in \mathbb {N}^\rhd$ for which $(\mathbf{M}_1^\rhd )_{ss}>0$ (see e.g., Woess 2009). This is the case for $s_\mathrm{{min}}$ when $\max \{\mu _\mathrm{{sdel}},\mu _\mathrm{{ldel}}\}>0$ as all the transitions going below $s_\mathrm{{min}}$ are rewired to $s_\mathrm{{min}}$.$\square$

#### **Property 9**

If $$s_0 \ge s_\mathrm{{min}},\, \forall n \ge 1$$,

$$\begin{aligned} \mathrm{Pr}_{s_0}\left[ \bigcap \limits _{k=1}^{n} ( S^\rhd _k > s_\mathrm{{min}})\right] = \mathrm{Pr}_{s_0}\left[ \bigcap \limits _{k=1}^{n} ( S_k > s_\mathrm{{min}})\right] \end{aligned}$$

This property is worth noting but it is rather obvious from the rewiring: it involves only transitions that have the same probabilities on both sides at any step. It comes from the fact that for large genomes $(\mathbb {N}^\rhd ,\mathbf{M}_1^\rhd )$ behaves exactly like $(\mathbb {N},\mathbf{M}_1)$.

We arrive to the property that we originally wanted to show and that follows from Property 7.

#### **Property 10**

Let $$s_0 \in \mathbb {N}$$ and $$s_0^\rhd = \max \{s_0,s_\mathrm{{min}}\}$$.

$$\begin{aligned} \forall n \ge 0, \forall s \ge s_\mathrm{{min}}, \quad \mathrm{Pr}_{s_0}\left[ S_n \le s\right] \ge \mathrm{Pr}_{s_0^\rhd }\left[ S^\rhd _n \le s\right] \end{aligned}$$

In particular $$\mathrm{Pr}_{s_0}\left[ S_n \le s_\mathrm{{min}}\right] \ge \mathrm{Pr}_{s_0^\rhd }\left[ S^\rhd _n = s_\mathrm{{min}}\right] $$.

#### *Proof*

The proof is by induction. As $s^\rhd _0 \ge s_0$, the property is obvious for $n=0$. We suppose that the property holds for some $n\ge 0$. Let $s \ge s_\mathrm{{min}}$. We need to show that $\mathrm{Pr}_{s_0}\left[ S_{n+1}\le s \right] - \mathrm{Pr}_{s_0^\rhd }\left[ S^\rhd _{n+1}\le s\right] \ge 0$. We will use conditioning over the distributions at step $n$. If we want the result to be meaningful, we need to compare the distribution given by the same quantiles of $S_n$ and $S_n^\rhd$. $\forall x \in [0,1]$, let $q_x = \min \{k\in \mathbb {N}: \mathrm{Pr}_{s_0}\left[ S_n\le k\right] \ge x\}$, the quantile function of $S_n$. Because the genome size is bounded for every $n$ (duplication at most double the genome size), $q_1$ is well defined and, as $\mathbb {N}$ is discrete, $q_x$ is piecewise constant. We write the conditioning according to the quantile function as

$$\begin{aligned} \forall x \in [0,1], \quad h(x) = \;&\mathrm{Pr}_{s_0}\left[ S_{n+1}\le s | S_n < q_x \right] \mathrm{Pr}_{s_0}\left[ S_n < q_x\right] \\&+ \mathrm{Pr}_{}\left[ S_{n+1}\le s | S_n = q_x \right] (x - \mathrm{Pr}_{s_0}\left[ S_n < q_x \right] ) \end{aligned}$$

We sum contributions up to the state preceding $q_x$ and $x$ controls the amount of contributions coming from $q_x$. When $x=\mathrm{Pr}_{s_0}\left[ S_n\le q_x\right]$ is reached, $q_x$ has to be increased. We have that $h(0)=0,\, h(1)=\mathrm{Pr}_{s_0}\left[ S_{n+1}\le s \right]$ and $h$ is continuous and piecewise linear. The points were $h$ cannot be differentiated are the points $\{ \mathrm{Pr}_{s_0}\left[ S_n\le k\right] , k \in \mathbb {N}\}$, because the value of $q_x$ changes. Because genome size is bounded, this set is finite. Similarly, we define $q^\rhd _x$ and $h^\rhd$, using $S^\rhd _n$ and $\mathbb {N}^\rhd$. We have $q_x \le q^\rhd _x$ by our induction hypothesis and, when the derivative is defined (as $q_x$ and $q_x^\rhd$ are constant in this case)

$$\begin{aligned} (h - h^\rhd )'(x) = \mathrm{Pr}_{}\left[ S_{n+1}\le s | S_n = q_x \right] - \mathrm{Pr}_{}\left[ S^\rhd _{n+1}\le s | S^\rhd _n = q^\rhd _x \right] \end{aligned}$$

We apply Property 7, $(h - h^\rhd )'(x) \ge \mathrm{Pr}_{}\left[ S_{n+1}\le s | S_n {=} q^\rhd _x \right] - \mathrm{Pr}_{}\left[ S^\rhd _{n{+}1}\le s | S^\rhd _n {=} q^\rhd _x \right]$. By the definition of $S^\rhd ,\, \mathrm{Pr}_{}\left[ S_{n+1}\le s | S_n = q^\rhd _x \right] = \mathrm{Pr}_{}\left[ S^\rhd _{n+1}\le s | S^\rhd _n = q^\rhd _x \right]$, thus $(h - h^\rhd )'(x) \ge 0$. By integrating over $x \in [0,1]$, using the fact that the number of points where the derivative is not defined is finite and $h-h^\rhd$ is continuous everywhere, we get

$$\begin{aligned} (h - h^\rhd )(1)=\mathrm{Pr}_{s_0}\left[ S_{n+1} \le s\right] - \mathrm{Pr}_{s_0^\rhd }\left[ S^\rhd _{n+1} \le s\right] \ge 0 \end{aligned}$$

$$\square $$

#### *Remark 3*

The properties given in this subsection apply mostly to small genomes, the general idea being that if we know how $s_\mathrm{{min}}$ behaves, we have information about what happens below. Process $(\mathbb {N}^\rhd ,\mathbf{M}_1^\rhd )$ will be useful when it comes to showing that genomes starting below $s_\mathrm{{min}}$ partly stay below $s_\mathrm{{min}}$. It suffices to show it for $s_\mathrm{{min}}$ in $(\mathbb {N}^\rhd ,\mathbf{M}_1^\rhd )$: Property 10 shows that if we take some smaller starting point in $(\mathbb {N},\mathbf{M}_1)$, it is even worse, we have created a worst-case scenario.

#### *Remark 4*

Property 7, used to create the worst-case scenario, is in fact not necessary. We could have aggregated the states below $\tilde{s}$ in a different way, for example by monitoring all transitions starting below $\tilde{s}$ and creating a chimeric state based on the transition which go farthest above $\tilde{s}$ (there is only a finite number of transitions starting below $\tilde{s}$ so a worst transition is bound to exist). This would relax some hypotheses that are not necessary for the existence of the stationary distribution, e.g., the indel distribution could freely depend on the starting size $s_0$. However, Property 7 seems biologically plausible and is fulfilled for the distributions usually considered in the literature.

### Proof of Lemma 2

We now have all the tools to prove Lemma 2. According to the properties on large genomes, large genomes tend to shrink when $(2\log 2-1) \mu _\mathrm{{dup}}- \mu _\mathrm{{ldel}}<0$. We still have to show at what speed they get below threshold $\tilde{s}$ and that they tend to remain there asymptotically. By the properties on small genomes, we know that to analyze what happens below $\tilde{s}$ we can look at $\tilde{s}$ only thanks to $S^\rhd$ (Remark 3). Showing that genomes remain around $\tilde{s}$ can be seen from the return time viewpoint. If they indeed stay around this value, one expects that the return times are rather small, typically that their expected value is finite. We show that this is indeed the case and sufficient for our proof.

#### *Proof (of Lemma 2)*

- (a) is exactly Property 6.
- We proceed in four steps to show (b). Step 1 is dedicated to showing that large genomes asymptotically end up below $\tilde{s}$, step 2 that small genomes tend to remain small by analyzing what happens starting from $\tilde{s}$, step 3 logically concludes on what happens when genomes are small from the beginning and step 4 uses the conclusions of step 1 and 2 to show that large genomes asymptotically get below $\tilde{s}$ and stay there.
  1. Let $s_0 \ge \tilde{s}$. $\forall n \ge 0$, define $A_n = \bigcap _{0\le k \le n} ( S_k \ge \tilde{s})$, $p_n = \mathrm{Pr}_{s_0}\left[ A_n\right]$ and $e_n = \mathbb {E}_{s_0}\left[ \log (S_n)|A_n\right]$. Conditioning on the outcome at step $n+1$ yields
    $$\begin{aligned} \mathbb {E}_{s_0}\left[ \log (S_{n+1}) | A_n\right] =&\mathbb {E}_{s_0}\left[ \log (S_{n+1}) | A_{n+1}\right] \frac{p_{n+1}}{p_n} \\&+ \mathbb {E}_{s_0}\left[ \log (S_{n+1}) | A_{n} \cap (S_{n+1}<\tilde{s})\right] \\&\times \frac{\mathrm{Pr}_{s_0}\left[ A_{n} \cap (S_{n+1}<\tilde{s})\right] }{p_n} \end{aligned}$$
    As $p_n > 0$ and $\mathbb {E}_{s_0}\left[ \log (S_{n+1}) | A_{n} \cap S_{n+1}<\tilde{s}\right] \ge 0$ (as $\log$ here is a positive function, see Remark 1),
    $$\begin{aligned} e_{n+1} p_{n+1}&= \mathbb {E}_{s_0}\left[ \log (S_{n+1}) | A_{n+1}\right] p_{n+1} \le \mathbb {E}_{s_0}\left[ \log (S_{n+1}) | A_n\right] p_n \\&\le (e_n - \delta ) p_n \end{aligned}$$
    The last inequality follows from Property 6 (see Remark 2), combined with the Markov property. By induction, we get
    $$\begin{aligned} e_n p_n \le e_0 p_0 - \delta \sum \limits _{k=0}^{n-1}p_k = \log s_0 - \delta \sum \limits _{k=0}^{n-1}p_k \le \log s_0 - n \delta p_n \end{aligned}$$
    The last inequality results from the fact that the sequence $(p_n)_{n \in \mathbb {N}}$ is decreasing. Finally
    10 $$\begin{aligned} p_n \le \frac{\log s_0}{e_n + n \delta } \le \frac{\log s_0}{\log \tilde{s}+ n \delta }, \end{aligned}$$
    which concludes the proof of the first step. What is more, going back to the previous inequality, we get a bound on the partial sum
    $$\begin{aligned} \delta \sum \limits _{k=0}^{n}p_k \le \log s_0 - e_{n+1} p_{n+1} \le \log s_0 \end{aligned}$$
  2. We use Definition 5 with $s_\mathrm{{min}} = \tilde{s}$. Let $T$ be the time of first return to $\tilde{s}$ in $(\mathbb {N}^\rhd ,\mathbf{M}_1^\rhd )$. $\forall n \ge 0$, define $B_n = (S_0 \ge \tilde{s}) \cap \bigcap _{1\le k \le n} ( S_k > \tilde{s}) \subset A_n$. Note that $\mathrm{Pr}_{\tilde{s}}\left[ B_{0}\right] =1$ so that for $k\ge 1,\,\mathrm{Pr}_{\tilde{s}}\left[ T=k\right] = \mathrm{Pr}_{\tilde{s}}\left[ B_{k-1}\right] - \mathrm{Pr}_{\tilde{s}}\left[ B_k\right]$. By Property 9, $\forall s_0 \ge \tilde{s},\, \forall n \ge 1$,
    11 $$\begin{aligned} \mathrm{Pr}_{s_0}\left[ \bigcap \limits _{k=1}^n ( S^\rhd _k > \tilde{s})\right] = \mathrm{Pr}_{s_0}\left[ \bigcap \limits _{k=1}^n ( S_k > \tilde{s})\right] = \mathrm{Pr}_{s_0}\left[ B_n\right] \le \mathrm{Pr}_{s_0}\left[ A_n\right] = p_n\nonumber \\ \end{aligned}$$
    so $\mathrm{Pr}_{\tilde{s}}\left[ T < +\infty \right] = 1 - \mathrm{Pr}_{\tilde{s}}\left[ \bigcap _{k\ge 1} ( S^\rhd _k > \tilde{s})\right] \ge 1 - \lim _n p_n = 1$. This shows that $\tilde{s}$ is a recurring state in $(\mathbb {N}^\rhd ,\mathbf{M}_1^\rhd )$. What is more,
    $$\begin{aligned} \mathbb {E}_{\tilde{s}}\left[ T\right] = \sum _{k\ge 1} k \mathrm{Pr}_{\tilde{s}}\left[ T=k\right] = \sum _{k\ge 1} k \left( \mathrm{Pr}_{\tilde{s}}\left[ B_{k-1}\right] - \mathrm{Pr}_{\tilde{s}}\left[ B_k\right] \right) \end{aligned}$$
    We reorganize terms within the series by looking at the partial sums
    $$\begin{aligned}&\sum _{k = 1}^{n}k \mathrm{Pr}_{\tilde{s}}\left[ B_{k-1}\right] - \sum _{k = 1}^{n}k\mathrm{Pr}_{\tilde{s}}\left[ B_k\right] = \sum _{k = 0}^{n-1}(k+1) \mathrm{Pr}_{\tilde{s}}\left[ B_k\right] - \sum _{k = 1}^{n}k\mathrm{Pr}_{\tilde{s}}\left[ B_k\right] \\&\quad = \sum _{k = 0}^{n-1} \mathrm{Pr}_{\tilde{s}}\left[ B_k\right] - n \mathrm{Pr}_{\tilde{s}}\left[ B_n\right] \le \sum _{k = 0}^{n-1} p_k \le \frac{\log \tilde{s}}{\delta } \end{aligned}$$
    Where the last inequalities follow from (11) and the conclusion of step 1. From the partial sum, we can easily infer that $\mathbb {E}_{\tilde{s}}\left[ T\right] < +\infty$. This shows that $\tilde{s}$ is a positive recurrent state. $(\mathbb {N}^\rhd ,\mathbf{M}_1^\rhd )$ is thus irreducible, aperiodic (Property 8) and positive recurrent. The convergence theorem for positive recurrent chains shows that there is a unique asymptotic stationary distribution and $\lim _n \mathrm{Pr}_{}\left[ S^\rhd _n = \tilde{s}\right] = 1 / \mathbb {E}_{\tilde{s}}\left[ T\right] > 0$ (see Woess 2009). What is more, $\forall n \ge 0$, $\mathrm{Pr}_{\tilde{s}}\left[ S^\rhd _n = \tilde{s}\right] > 0$ as $(\mathbf{M}_1^\rhd )_{\tilde{s}\tilde{s}}>0$. These two remarks imply that the set $\{\mathrm{Pr}_{\tilde{s}}\left[ S^\rhd _n = \tilde{s}\right] , n\in \mathbb {N}\}$ is inferiorly bounded by some $\varepsilon >0$.
  3. Property 10 allows us to apply the conclusion of the last step to $(\mathbb {N},\mathbf{M}_1)$
    $$\begin{aligned} \forall s_0 \le \tilde{s}, \forall n \in \mathbb {N}, \quad \mathrm{Pr}_{s_0}\left[ S_n \le \tilde{s}\right] \ge \mathrm{Pr}_{\tilde{s}}\left[ S^\rhd _n = \tilde{s}\right] \ge \varepsilon \end{aligned}$$
    This proves inequality (b)i. of the lemma. As the chain is time homogeneous, this can be generalized as
    12 $$\begin{aligned} \forall s \le \tilde{s}, \forall k \ge 0, \forall n \ge 0, \quad \mathrm{Pr}_{}\left[ S_{n+k} \le \tilde{s}| S_k = s\right] \ge \varepsilon \end{aligned}$$
    When we applied Property 8 in the previous step, we neglected the case $\mu _\mathrm{{dup}}=\mu _\mathrm{{ins}}=0$. However, in this case, the inequalities presented in this step are obvious with $\varepsilon = 1$.
  4. Let $s_0 > \tilde{s}$. We compute $\mathrm{Pr}_{s_0}\left[ S_n \le \tilde{s}\right]$ by partitioning over the time of first passage below $\tilde{s}$,
    $$\begin{aligned} \mathrm{Pr}_{s_0}\left[ S_n \le \tilde{s}\right] =&\sum _{k\ge 1} \mathrm{Pr}_{s_0}\left[ S_n \le \tilde{s}| S_k \le \tilde{s}, S_{k-1}>\tilde{s}, \ldots , S_0>\tilde{s}\right] \\&\times \mathrm{Pr}_{s_0}\left[ S_k \le \tilde{s},S_{k-1}>\tilde{s}, \ldots , S_0>\tilde{s}\right] \end{aligned}$$
    Note that all terms $k>n$ are zero. If we use the definiton of $B_k$ and relation (12) (applying Remark 2) when $n \ge 1$
    $$\begin{aligned} \mathrm{Pr}_{s_0}\left[ S_n \le \tilde{s}\right] \ge \varepsilon \sum _{k=1}^{n} (\mathrm{Pr}_{s_0}\left[ B_{k-1}\right] -\mathrm{Pr}_{s_0}\left[ B_k\right] ) = \varepsilon (1-\mathrm{Pr}_{s_0}\left[ B_n\right] ) \end{aligned}$$
    We finally apply relation (11) and (10)
    $$\begin{aligned} \mathrm{Pr}_{s_0}\left[ S_n \le \tilde{s}\right] \ge \varepsilon (1-p_n) \ge \varepsilon \left( 1 - \frac{\log s_0}{\log \tilde{s}+ n \delta }\right) \end{aligned}$$
    This proves inequality (b)ii. of the lemma. The relation for $n=0$ is trivial as the right-hand side is negative.

$$\square $$

## Appendix 2: Proofs for the Continuous Case

### *Proof*

(Proof (of Property 4)) The most important point is the change of summation subset when summing over the Poisson distribution, as in

$$\begin{aligned}&\sum _{n\ge 0} n \frac{((\mu _\mathrm{{ldel}}+\mu _\mathrm{{dup}})s_0)^n}{n!}e^{-(\mu _\mathrm{{ldel}}+\mu _\mathrm{{dup}})s_0} \\&\quad = (\mu _\mathrm{{ldel}}+\mu _\mathrm{{dup}})s_0 e^{-(\mu _\mathrm{{ldel}}+\mu _\mathrm{{dup}})s_0} \sum _{n\ge 1} \frac{((\mu _\mathrm{{ldel}}+\mu _\mathrm{{dup}})s_0)^{n-1}}{(n-1)!} \\&\quad = (\mu _\mathrm{{ldel}}+\mu _\mathrm{{dup}})s_0 \end{aligned}$$

In the following we define $\hbox {P}\Sigma \left[ \cdot \right]$ as the operator for the Poisson summation (more precisely, $\hbox {P}\Sigma \left[ \right]$ is the expected value with respect to the Poisson distribution). For example, we write the relation above as $\hbox {P}\Sigma \left[ n\right] =(\mu _\mathrm{{ldel}}+\mu _\mathrm{{dup}})s_0$. Similarly, we can show that $\hbox {P}\Sigma \left[ n(n-1)\right] =(\mu _\mathrm{{ldel}}+\mu _\mathrm{{dup}})^2s_0^2$. In the following, we allow ourselves to interchange the operators $\hbox {P}\Sigma \left[ \cdot \right]$ and $\mathbb {E}_{}\left[ \cdot \right]$, we will justify this at the end of the proof.

$$\begin{aligned} \mathbb {E}_{s_0}\left[ \log \hat{S}_f\right]&= \hbox {P}\Sigma \left[ \mathbb {E}_{s_0}\left[ \log \hat{S}_n\right] \right] = \log s_0 + \mathbb {E}_{}\left[ J_n\right] \hbox {P}\Sigma \left[ n\right] \\&= \log s_0 + s_0 ((2\log 2 -1 )\mu _\mathrm{{dup}}- \mu _\mathrm{{ldel}}). \end{aligned}$$

To simplify the computation of the second moment, we call $A = \mu _\mathrm{{ldel}}- (2\log 2 -1 )\mu _\mathrm{{dup}}$ and $B = \sqrt{2(\mu _\mathrm{{ldel}}+(1-\log 2)^2\mu _\mathrm{{dup}})}$. We have $\hbox {P}\Sigma \left[ n\mathbb {E}_{}\left[ J_n\right] \right] =-A s_0$ and $\hbox {P}\Sigma \left[ n\mathbb {E}_{}\left[ J_n^2\right] \right] =B^2 s_0$. The variance of $\hat{S}_f$ is given by the formula

$$\begin{aligned} \mathbb {E}_{s_0}\left[ \log ^2 \hat{S}_f\right] - \left( \mathbb {E}_{s_0}\left[ \log \hat{S}_f\right] \right) ^2 = \hbox {P}\Sigma \left[ \mathbb {E}_{s_0}\left[ \log ^2 \hat{S}_n\right] \right] -(\log s_0 - A s_0)^2 \end{aligned}$$

where

$$\begin{aligned} \mathbb {E}_{s_0}\left[ \log ^2 \hat{S}_n\right]&= \sigma ^2_{s_0}\left[ \log \hat{S}_n\right] +\left( \mathbb {E}_{s_0}\left[ \log \hat{S}_n\right] \right) ^2 = n \sigma ^2_{}\left[ J_n\right] + (\log s_0 + n \mathbb {E}_{}\left[ J_n\right] )^2 \\&= \log ^2 s_0 + 2\log s_0 n\mathbb {E}_{}\left[ J_n\right] + n(n-1)(\mathbb {E}_{}\left[ J_n\right] )^2 + n\mathbb {E}_{}\left[ J_n^2\right] \end{aligned}$$

We apply the Poisson summation and use the various definitions and properties

$$\begin{aligned} \hbox {P}\Sigma \left[ \mathbb {E}_{s_0}\left[ \log ^2 \hat{S}_n\right] \right]&= \log ^2 s_0 - 2 \log s_0 A s_0 + A^2 s_0^2 + B^2 s_0\\&= (\log s_0 - A s_0)^2 + B^2 s_0 \end{aligned}$$

We deduce

$$\begin{aligned} \sigma _{s_0}\left[ \log \hat{S}_f\right] = \sqrt{B^2 s_0} = \sqrt{s_0} \sqrt{2(\mu _\mathrm{{ldel}}+(1-\log 2)^2\mu _\mathrm{{dup}})} \end{aligned}$$

Interchanging $\hbox {P}\Sigma \left[ \cdot \right]$ and $\mathbb {E}_{}\left[ \cdot \right]$ is legitimate as an application of Fubini-Tonelli’s theorem. As $\hbox {P}\Sigma \left[ \mathbb {E}_{s_0}\left[ \log ^2 \hat{S}_n\right] \right] < +\infty$, the conditions of the theorem are met for the second moment, and as, asymptotically, $|x|<x^2$, this extends to the first moment.$\square$

### *Proof (of Lemma 3)*

We have

$$\begin{aligned} Q_k'(s_0) = Q_k(s_0) \left( \frac{1}{s_0} - A + \frac{kB}{2\sqrt{s_0}}\right) = Q_k(s_0) \frac{2-2A s_0+kB\sqrt{s_0}}{2s_0} \end{aligned}$$

The sign of the derivative is determined by $2-2A s_0+kB\sqrt{s_0}$ which cancels for a unique value

$$\begin{aligned} \sqrt{s_0} = \frac{kB+\sqrt{k^2B^2+16A}}{4A} \end{aligned}$$

(because $A >0$). Squaring this relation yields $s_{0}^{{\max },(k)}$. As the main coefficient $-2A$ is negative, the derivative is positive below $s_{0}^{{\max },(k)}$ and negative above.

To obtain $s_\mathrm{{fixed}}^{(k)}$, we compute $Q_k(s_0)=s_0$ which yields $\sqrt{s_{0}} = kB/A$ and, as $A>0$, the value of $s_\mathrm{{fixed}}^{(k)}$ is immediate. What is more

$$\begin{aligned} Q_k'(s_\mathrm{{fixed}}^{(k)}) = \frac{Q_k(s_\mathrm{{fixed}}^{(k)})}{2s_\mathrm{{fixed}}^{(k)}}\left( 2 - 2 k^2\frac{B^2}{A} + k^2 \frac{B^2}{A} \right) = \frac{1}{2}\left( 2 - k^2\frac{B^2}{A} \right) \end{aligned}$$

We can rewrite the ratio $B^2/A$ as

$$\begin{aligned} \frac{B^2}{A} = 2\times \frac{(\log 2 - 1)^2\mu _\mathrm{{dup}}+\mu _\mathrm{{ldel}}}{\mu _\mathrm{{ldel}}- (2\log 2 -1 )\mu _\mathrm{{dup}}} = 2\times \frac{1+(\log 2 - 1)^2(\mu _\mathrm{{dup}}/\mu _\mathrm{{ldel}})}{1 - (2\log 2 -1 )(\mu _\mathrm{{dup}}/\mu _\mathrm{{ldel}})} \ge 2 \end{aligned}$$

For the last inequality, we show that the ratio is equal to 2 for $\mu _\mathrm{{dup}}/\mu _\mathrm{{ldel}}=0$ and increases to infinity when $\mu _\mathrm{{dup}}/\mu _\mathrm{{ldel}}$ tends to $1/((2\log 2 -1))$. We supposed that $k \ge 1$, thus $Q'_k(s_\mathrm{{fixed}}^{(k)})\le 0$. As the subset where the derivative is negative is $\{s\ge s_{0}^{{\max },(k)}\}$, necessarily $s_\mathrm{{fixed} }^{(k)}\ge s_{0}^{{\max },(k)}$.$\square$
